# Influence of non-pharmaceutical COVID-19 interventions on speed-related and alcohol-related traffic injuries in five cities of the province of Québec, Canada

**DOI:** 10.1136/ip-2024-045481

**Published:** 2025-01-02

**Authors:** José Ignacio Nazif-Munoz, Brice Batomen, Thomas Gordon Brown, Camila Corrêa Matias Pereira, Claude Giroux, Asma Mamri, Vahid Najafi Moghaddam Gilani, Marie Claude Ouimet, Cynthia Paquet, Émilie Turmel, Ward Vanlaar

**Affiliations:** 1 Université de Sherbrooke, Longueuil, Quebec, Canada; 2 Dalla Lana School of Public Health, Division of Epidemiology, University of Toronto, Toronto, Ontario, Canada; 3 Université de Sherbrooke, Sherbrooke, Quebec, Canada; 4 SAQ, Montreal, Quebec, Canada; 5 Medicine and Health Science, University of Sherbrooke Longueuil Campus, Longueuil, Quebec, Canada; 6 Traffic Injury Research Foundation, Ottawa, Ontario, Canada

**Keywords:** COVID-19, Alcohol, Speed, Time series

## Abstract

**Introduction:**

Understanding the impact of non-pharmaceutical COVID-19 interventions (NPIs) on road safety has become increasingly important to uncover the unintended consequences of the pandemic. This study explores how NPIs influenced alcohol-related and speed-related traffic collisions, including fatalities and serious injuries, in five cities of the province of Québec, Canada: Montréal, Québec, Laval, Longueuil and Sherbrooke.

**Methods:**

We performed Poisson interrupted time-series analyses using daily traffic fatality and injury data from 2015 to 2022, to assess the change in rate expressed per 10 000 population. A Québec COVID-19 NPIs Index was applied, incorporating 58 interventions enacted from March 2020 to March 2022 in these cities. We accounted for weather conditions and seasonal patterns and divided the pandemic period into four semesters to better understand changes over time.

**Results:**

The analysis revealed a nuanced interaction between NPIs and road safety. Alcohol-related injuries decreased during stringent NPIs, particularly in Montréal, Québec city and Longueuil. In contrast, the effects on speed-related incidents were mixed, with Montréal and Laval, showing increases and the other three cities displaying no meaningful changes across the four semesters.

**Conclusions:**

These findings highlight the necessity for ad hoc road safety strategies that address specific patterns of alcohol consumption and speeding risks during future pandemic-related disruptions.

**WHAT IS ALREADY KNOWN ON THIS TOPIC**

**WHAT THIS STUDY ADDS**

- Data on alcohol-related and speed-related traffic injuries are presented for five cities in Quebec across four semesters during the initial 2 years of the COVID-19 pandemic. Significant reductions in alcohol-related light injuries were observed in Montréal during semesters 1 (incidence rate ratio (IRR): 0.77, 95% CI: 0.66 to 0.88), 2 (IRR: 0.68, 95% CI: 0.55 to 0.83) and 3 (IRR: 0.86, 95% CI: 0.72 to 1.01); in Québec city during semester 1 (IRR: 0.79, 95% CI: 0.60 to 1.03); and in Longueuil during semester 1 (IRR: 0.62, 95% CI: 0.43 to 0.88). Conversely, Montréal experienced a 20% increase in speed-related light injuries during semester 1 (IRR: 1.20, 95% CI: 1.00 to 1.35).

**HOW THIS STUDY MIGHT AFFECT RESEARCH, PRACTICE OR POLICY**

- Enhanced speeding enforcement programmes and city-specific strategies considering rapid adjustments to enforcement and public health messaging based on real-time data could improve overall traffic safety during future disruptions.

## Introduction

 The declaration of COVID-19 as a global health emergency by the WHO in January 2020 led to the rapid implementation of various non-pharmaceutical public health interventions (NPIs) by governments worldwide. These measures, which included closures of schools, non-essential businesses, public events and the promotion of remote work and curfews, had a significant impact on transportation and mobility.^1–5^ However, the effect of these NPIs on road safety, particularly concerning alcohol and speeding as key risk factors, has not been thoroughly examined.

In Canada, different provinces, territories and municipalities adopted varying approaches to NPIs,^6^ with Québec standing out as an early and rigorous enforcer, being the first province to declare a state of emergency.^7^ Québec’s major cities—Montréal, Longueuil, Québec City, Laval and Sherbrooke—exhibited diverse responses to these interventions, making them important case studies for examining the impact of NPIs on road safety.^8^

While existing literature has noted a general decline in traffic collisions during the pandemic due to reduced mobility,^9–11^ the specific influence of alcohol consumption and speeding on road safety during this period requires further exploration. Reduced traffic volume jointly with decreases in law enforcement might have led to increased speeding,^12^ while the stress and anxiety associated with the pandemic could have contributed to higher rates of alcohol consumption that may have led to increased impaired driving due to increases in alcohol consumption.^13^ In addition, adjustments to COVID-19 NPIs, such as restrictions on alcohol sales or limited hours for alcohol consumption, may have also contributed to a decrease in alcohol-related injuries.^14^ The combination of these factors makes it challenging to assess their overall impact on road safety.

To address these knowledge gaps, this study analyses the variations in the timing, nature and types of NPI implementations across five Québec cities (ie, Montréal, Québec City, Laval, Longueuil and Sherbrooke) with a specific focus on alcohol-related and speed-related traffic injuries. By examining these factors, the study aims to provide a more accurate understanding of how NPIs affect road safety, particularly regarding the risks associated with alcohol and speeding.

This research is particularly important as it seeks to fill gaps in our understanding of road safety during the COVID-19 pandemic by focusing on the specific risk factors of alcohol consumption and speeding. It examines the relationship between COVID-19 NPIs and road safety outcomes, with an emphasis on traffic collisions, lightly injured and killed and severely injured (KSI) in the context of these risk factors. The study’s findings will contribute to the broader discussion on how public health measures can be balanced with maintaining road safety, particularly in the face of future pandemic emergencies.

## Methods

### Study design

In this research, we examine the effects of various COVID-19 NPIs on alcohol-related and speeding-related light injuries, as well as KSI cases in five Québec cities: Montréal, Sherbrooke, Laval, Longueuil and Québec city. We use an interrupted time-series design, with our analysis period spanning from 1 January 2015 to 11 March 2022.

### Data sources

We obtained crash data from the Société de l’assurance automobile du Québec (SAAQ),^15^ which included factors associated with crashes, such as speeding and impaired driving. We also incorporated population data from the Institut de la statistique du Québec,^16^ weather information from Environment Canada^17^ and multiple sources were identified to determine the duration and type of NPIs across the five cities.

Patients or the public were not involved in the design, or conduct, or reporting, or dissemination plans of our research.

### Dependent variables

Four dependent variables were used to assess the potential effects of NPIs on four road safety outcomes: (1) the total number of lightly injured individuals in alcohol-related crashes, (2) the total number of KSI in alcohol-related crashes, (3) the total number of lightly injured individuals in speeding-related crashes and (4) the total number of KSI in speeding-related crashes. The SAAQ data set includes information, such as (1) the date of the crash, (2) the city where the crash occurred, (3) the type of injury (either light or KSI) reported and (4) factors associated with the crash (eg, speeding, alcohol consumption, fatigue). By using the crash date, we identified whether the outcome happened before or after the NPIs. The city identification code allowed us to identify all injuries and KSIs within the five cities. The factors associated with crashes allowed to identify whether the crashes were linked to speeding and/or alcohol consumption. The denominator used was population per year and per city.

### Intervention: the Québec Non-Pharmaceutical Interventions Index

We use multiple sources, such as the Canadian COVID-19 Intervention Timeline Tool developed by the Canadian Institute for Health Information, to identify these interventions. Although this tool provided a structured way to organise COVID-19 NPIs based on Government of Canada data, interventions, however, were categorised at provincial or territory levels. To accurately capture city-specific and temporal variations, we complement these data with information from official websites, public health organisations and media reports, within the province of Québec. Details of this methodology are provided in Mamri *et al*.^8^

After identifying the interventions, we created the Québec Non-Pharmaceutical Interventions Index (QCnPI-Index), which is organised into three main categories: travel, physical distancing and closure/opening. The Travel category governed movement between locations, physical distancing imposed distancing requirements and closure/opening involved the suspension or resumption of operations. This framework resulted in 58 interventions, categorised by their duration on a daily basis. The QCnPI-Index values range from 0 (indicating no interventions, particularly before 12 March 2020) to 0.56 (the highest recorded value). A higher score reflects a more extensive and intensive implementation of NPIs in each city, while lower scores indicate fewer measures and a score of 0 represents an absence of interventions.

To facilitate interpretation, the index was scaled by multiplying it by 10.

To analyse the varying associations of the QCnPI-Index over time, we focused on the period from when COVID-19 was declared a pandemic in Québec (12 March 2020 to 11 March 2022). This period is divided into four semesters: Semester 1 (12 March 2020 to 11 September 2020); semester 2 (12 September 2020 to 11 March 2021); semester 3 (12 March 2021 to 11 September 2021); and semester 4 (12 September 2021 to 11 March 2022). To examine whether the impact of the QCnPI-Index on each selected outcome varied by city, we first compared each of the four semesters individually to the prepandemic period (1 January 2015 to 11 March 2020). Finally, we compared results across the semesters with each other.

### Control variables

Due to the complex nature of crashes involving alcohol and speeding, we considered various factors to better adjust the association of the QCnPI-Index with our selected outcomes. These factors included the implementation of the cannabis law in October 2018,^18 19^ seasonal variations using Fourier terms,^20^ fluctuations in temperature,^21^ lags^22^ and temporal trends.^23^

### Statistical analysis

Due to the non-negative integer nature of road traffic crash count data, we used generalised linear models, particularly Poisson regression to assess how the introduction of NPIs, as measured by the QCnPI-Index, related to alcohol and speeding outcomes. Our models assumed that the conditional distribution of the count of alcohol’s or speed’s KSI or light injuries (y) in city (c) in day (d) was:


(1) $$Poisson:y_{cd}∼Poisson(\mu_{cd})$$

Generalised estimation equations with robust SEs, using lags to account for possible overdispersion and data dependency were performed.^24^ The statistical Poisson models follow the following equations:


(2) $$Log(\lambda_{cd})=X_{cd}\beta$$

(3) $$Log(U_{cd}/Pop_{cd})=X_{cd}\beta$$

(4) $$\begin{array}{l} Log(U_{cd})=log(Pop_{cd})+X\beta_{0}+\beta_{1}time_{cd}+\beta_{2}cannabis-law_{cd}\\ \;+\;\beta_{3}temp_{cd}+\beta_{4}sin_{cd}+\beta_{5}sin_{cd}+\beta_{6}cos_{cd}+\beta_{7}cos_{cd}\\ \;+\;\beta_{8}QCnPI-Index_{cd} \end{array}$$

Where:

λ_cd_=rate of events of alcohol-related and speeding-related light injuries or KSI in city c in day d,

Pop_cd_=population modelled as an offset in city c in day d,

β_0_ is the baseline level of the series in the log scale,

β_1_ is the baseline trend of the outcomes in the log scale,

β_2_ is the change in the level of log of the outcomes in the day following the introduction of the Cannabis law,

β_3_ is the change associated with the average of daily temperature,

β_4-7_ are sine and cosine pair of functions to smooth the periodic structure observed in the outcomes,^25^ including seasonality,

β_8_ represents the QCnPI-Index, acting as a proxy for a function due to the evolving nature of NPIs throughout the four semesters.

We present a total of 80 models, each representing a distinct combination of factors: (1) Five cities: Montréal, Québec, city Sherbrooke, Laval, Longueuil, and. (2) Four outcomes: alcohol-related light injuries and KSI, and speeding-related light injuries and KSI. (3) Four postperiods as previously described. Each model aimed to capture the unique dynamics of NPIs on alcohol and speeding outcomes. Zero-inflated Poisson statistical analyses as well as Poisson statistical analyses with traffic flow and congestion as two different denominators, were also performed, and results were robust to these model specifications. (Results are available in online supplemental tables S1–S8 of the supplementary material). To assess if incidence rate ratios (IRRs) differ significantly across semesters, we use the Wald test and calculate their respective Z-score for comparison.^26^ (Results are available in online supplemental tables S9–S10). Comprehensive results of Barlett and Ljun-Box tests^27 28^ to assess autocorrelation are provided in online supplemental tables S11–S12. All analyses were conducted using Stata V.17.^29^

## Results

Figure 1 depicts monthly changes over time of each outcome per city for the complete period of analysis (1 January 2015 to 11 March 2022). Table 1 displays the rates per 10 000 population of each outcome and the averages of the QCnPI-Index per semester. During the prepandemic period Longueuil exhibited the highest rate of alcohol-related and speed-related light injuries, whereas alcohol-KSI and speed-KSI were relatively similar across the five cities, with the exception of Sherbrooke where its speed-KSI was the lowest. During semesters 1 and 2, we observed lower levels of both alcohol-related and speeding-related crashes across all cities. In semester 3, we observed increases in both alcohol and speeding-related incidents in most cities. Following the peaks in semester 3, most cities exhibited a decrease in incidents, though the levels did not always return to the levels seen in semesters 1 and 2. While the general trend of a peak in semester 3 followed by a decline in semester 4 is observed, Sherbrooke showed unique patterns with increases continuing into semester 4, particularly in both light injuries’ outcomes. Overall, the trends suggest that during the pandemic alcohol-related and speed-related crashes had varying patterns of fluctuation and stabilisation across different cities. Finally, the averages of the QCnPI-Index peaked in semester 2 for all cities, except for Sherbrooke, which saw its highest value in semester 1. Conversely, all cities recorded their lowest values in semester 4, coinciding with the province achieving a consensus on the measures that should be implemented across cities.

**Figure 1:**
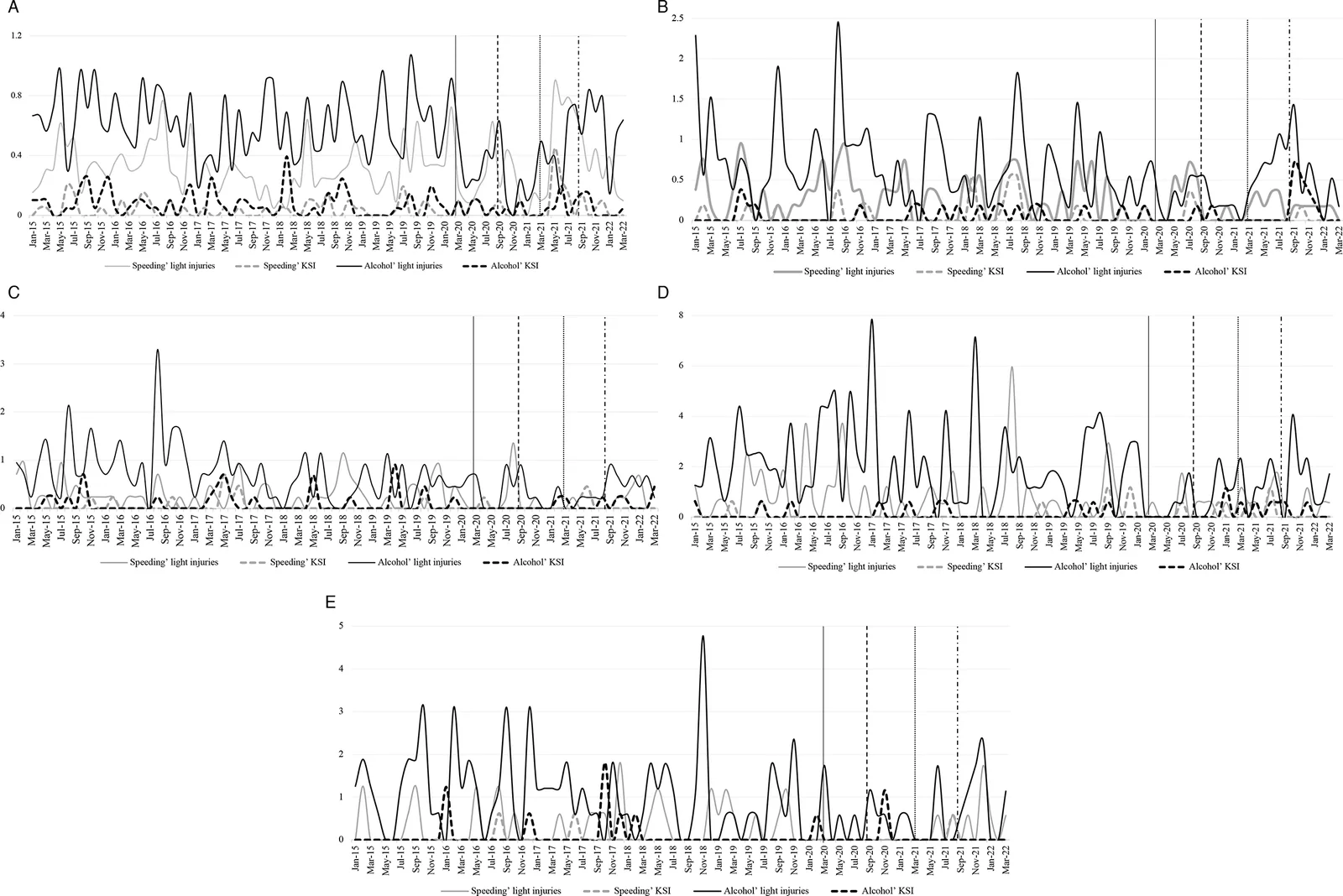
(A) Monthly rate of speeding light-injuries, speeding KSI, alcohol light-injuries and Alcohol KSI, per 100 000 population, January 2015 to March 2022, Montréal (B). Rate of speeding light-injuries, speeding KSI, alcohol light-injuries and alcohol KSI, per 100 000 population, January 2015 to March 2022, Québec (C). Rate of speeding light-injuries, speeding KSI, alcohol light-injuries and alcohol KSI, per 100 000 population, January 2015 to March 2022, Laval (D). Rate of Speeding light-injuries, speeding KSI, alcohol light-injuries and alcohol KSI, per 100 000 population, January 2015 to March 2022, Longueuil (E). Rate of Speeding light-injuries, speeding KSI, alcohol light-injuries and alcohol KSI, per 100 000 population, January 2015 to March 2022, Sherbrooke. KSI, killed and severely injured.

**Table 1:** Rates per 10 000 population per outcome and average of the QCnPI-Index during prepandemic and the four pandemic periods in each city

City	Population*	Outcome	Prepandemic period 1 January 2015 to 11 March 2020	Semester 1 12 March 2020 to 11 September 2020	Semester 2 12 September 2020 to 11 March 2021	Semester 3 12 March 2021 to 11 September 2021	Semester 4 12 September 2021 to 11 March 2022
Montréal	1 762 949	Alcohol’ light injuries	4.38	0.24	0.13	0.48	0.26
		Alcohol’ KSI	0.54	0.03	0.02	0.08	0.00
		Speeding’ light injuries	2.25	0.18	0.14	0.48	0.15
		Speeding’ KSI	0.27	0.04	0.01	0.11	0.02
		**Total**	**7.44**	**0.49**	**0.29**	**1.16**	**0.43**
		QCnPI-Index’s average†	**0.00**	**0.31 (0.02, 0.41**)	**0.35 (0.21, 0.56**)	**0.25 (0.06, 0.44**)	**0.14 (0.07, 0.30**)
Québec city	549 459	Alcohol’ light injuries	4.06	0.18	0.11	0.62	0.16
		Alcohol’ KSI	0.33	0.02	0.02	0.13	0.05
		Speeding’ light injuries	1.82	0.22	0.02	0.18	0.07
		Speeding’ KSI	0.33	0.05	0.00	0.02	0.00
		**Total**	**6.72**	**0.47**	**0.15**	**0.95**	**0.29**
		QCnPI-Index’s average†	**0.00**	**0.31 (0.02, 0.41**)	**0.32 (0.18, 0.54**)	**0.23 (0.06, 0.43**)	**0.14 (0.07, 0.30**)
Laval	443 192	Alcohol’ light injuries	4.47	0.18	0.18	0.25	0.25
		Alcohol’ KSI	0.59	0.00	0.02	0.07	0.07
		Speeding’ light injuries	1.69	0.16	0.02	0.05	0.14
		Speeding’ KSI	0.23	0.02	0.00	0.07	0.00
		**Total**	**6.97**	**0.36**	**0.23**	**0.43**	**0.45**
		QCnPI-Index’s average†	**0.00**	**0.31 (0.02, 0.41**)	**0.34 (0.19, 0.56**)	**0.25 (0.06, 0.44**)	**0.14 (0.07, 0.30**)
Longueuil	254 483	Alcohol’ light injuries	8.49	0.20	0.35	0.83	0.39
		Alcohol’ KSI	0.43	0.00	0.08	0.20	0.04
		Speeding’ light injuries	3.07	0.16	0.12	0.35	0.12
		Speeding’ KSI	0.28	0.04	0.04	0.12	0.00
		**Total**	**12.26**	**0.39**	**0.59**	**1.49**	**0.55**
		QCnPI-Index’s average†	**0.00**	**0.33 (0.02, 0.41**)	**0.34 (0.21, 0.56**)	**0.20 (0.06, 0.38**)	**0.14 (0.07, 0.30**)
Sherbrooke	172 950	Alcohol’ light injuries	6.34	0.30	0.30	0.36	0.54
		Alcohol’ KSI	0.54	0.00	0.12	0.00	0.00
		Speeding’ light injuries	1.91	0.00	0.00	0.18	0.30
		Speeding’ KSI	0.12	0.00	0.00	0.06	0.00
		**Total**	**8.91**	**0.30**	**0.42**	**0.60**	**0.84**
		QCnPI-Index’s average†	**0.00**	**0.32 (0.02, 0.41**)	**0.31 (0.19, 0.55**)	**0.19 (0.06, 0.37**)	**0.14 (0.07, 0.30**)

* Population values correspond to the year 2022; in online supplemental table S9 are depicted all populations values per city per year.

† Min and max values in parentheses.

KSI, killed or severely injured.; QCnPI, Québec Non-Pharmaceutical Interventions Index.

### The impact of the QCnPI-Index on alcohol light injuries and KSI

Table 2 shows the associations between the QCnPI-Index, across the four pandemic semesters and the rates of alcohol-related injuries and KSI, respectively. Online supplemental figure S1–S5 illustrate the IRR per city for alcohol-related crashes. The QCnPI-Index showed varied associations with alcohol-related injuries across cities. There were considerable decreases in alcohol-related light injuries in Montréal in semesters 1 (IRR: 0.77, 95% CI: 0.66 to 0.88), 2 (IRR: 0.68, 95% CI: 0.55 to 0.83) and 3 (IRR: 0.86, 95% CI: 0.72 to 1.01), in Québec city in semesters 1 (IRR: 0.79, 95% CI: 0.60 to 1.03) and 2 (IRR: 0.67, 95% CI: 0.44 to 0.99) and in Longueuil in semester 1 (IRR: 0.62, 95% CI: 0.43 to 0.88). No changes in alcohol-related KSI outcomes were observed across any city during the four post-pandemic periods.

**Table 2:** Associations between the QCnPI-Index and alcohol’ light injury and KSI rates per 100 000 population, 1 January 2015 to 19 March 2022

City	Outcome	Semester 1 12 March 2020 to 11 September 2020 *			Semester 2 12 September 2020 to 11 March 2021*			Semester 3 12 March 2021 to 11 September 2021*			Semester 4 12 September 2021 to 11 March 2022*		
		IRR	95% CI		IRR	95	% CI	IRR	95	% CI	IRR	95	% CI
Montréal	Alcohol’ light Injuries	**0.77**	**0.66**	**0.88**	**0.68**	**0.55**	**0.83**	0.86	0.72	1.01	0.81	0.57	1.13
	Alcohol’ KSI	0.90	0.60	1.34	0.82	0.49	1.35	1.00	0.72	1.50	1.00	0.36	2.67
Québec city	Alcohol’ light injuries	0.79	0.60	1.03	**0.67**	**0.44**	**0.99**	1.10	0.86	1.39	0.75	0.36	1.55
	Alcohol’ KSI	0.72	0.31	1.65	1.00	0.69	1.41	0.70	0.31	1.55	1.00	0.33	2.89
Laval	Alcohol’ light injuries	0.82	0.59	1.13	0.80	0.52	1.21	0.87	0.56	1.32	1.40	0.76	2.37
	Alcohol’ KSI	1.00	0.62	1.50	1.00	0.54	1.99	0.64	0.19	2.12	0.85	0.13	5.31
Longueuil	Alcohol’ light injuries	**0.62**	**0.43**	**0.88**	0.92	0.71	1.18	0.91	0.60	1.37	0.67	0.27	1.61
	Alcohol’ KSI	1.00	0.65	1.44	1.30	0.83	2.15	1.30	0.67	2.68	0.79	0.13	4.77
Sherbrooke	Alcohol’ light injuries	0.79	0.54	1.15	0.91	0.61	1.34	0.97	0.53	1.75	0.78	0.28	2.14
	Alcohol’ KSI	1.00	0.70	1.30	1.00	0.99	1.00	0.97	0.54	1.70	1.00	0.40	2.23

Models are adjusted for lags, time trends, daily temperature average, the Cannabis Act and sine and cosine pair of functions. The autocorrelation and partial autocorrelation information, and Ljung-Box and Bartlett tests values for each model are available online in online supplemental tables S11–S20 and online supplemental table 8, respectively. Bold outcomes indicate p<0.05.

* The QCnPI-Index was rescaled to facilitate the interpretation of the outcomes.

IRR, incidence rate ratios; KSI, killed or severely injured; QCnPI, Québec Non-Pharmaceutical Interventions Index.

### The impact of the QCnPI-Index on speeding light injuries and severe injuries–fatalities

Table 3 presents the associations between the QCnPI-Index across the four pandemic semesters and the rates of speed-related injuries and KSI. Online supplemental figure S6–S10 depict the IRR per city for speed-related crashes. In general, no significant associations between the QCnPI-Index and speed-related injuries across these cities were observed. Nevertheless, in Montréal and Laval during semester 3, a 0.1 increase in the QCnPI-Index was associated with a 1% increase (IRR: 1.01, 95% CI: 1.00 to 1.02) in speed-related KSI rates. Furthermore, in Montréal during the same semester a 0.1 increase in the QCnPI-Index was associated with a 20% increase (IRR: 1.20, 95% CI: 1.00 to 1.35) in speed-related light injuries. While both lower bounds of the 95% CIs are 1.00 when rounded at two decimals, both are higher than 1.00.

**Table 3:** Associations between the QCnPI-Index and speeding’ light injuries and KSI rates per 100 000 population, 1 January 2015 to 19 March 2022

City	Outcome	Semester 1 12 March 2020 to 11 September 2020*			Semester 2 12 September 2020 to 11 March 2021*			Semester 3 12 March 2021 to 11 September 2021*			Semester 4 12 September 2021 to 11 March 2022*		
		IRR	95	% CI	IRR	95	% CI	IRR	95	% CI	IRR	95	% CI
Montréal	Speeding’ light injuries	0.87	0.73	1.02	0.82	0.67	1.00	**1.20**	**1.00**	**1.35**	1.20	0.83	1.80
	Speeding’ KSI	1.10	0.76	1.49	1.00	0.99	1.00	**1.01**	**1.00**	**1.02**	0.31	0.01	5.10
Québec city	Speeding’ light injuries	1.10	0.81	1.43	0.59	0.26	1.31	1.10	0.76	1.59	1.20	0.55	2.78
	Speeding’ KSI	1.10	0.64	1.99	1.00	0.64	1.52	0.68	0.14	3.29	1.00	0.25	3.79
Laval	Speeding’ light injuries	0.86	0.60	1.21	0.57	0.23	1.37	0.21	0.03	1.41	1.60	0.82	3.09
	Speeding’ KSI	1.50	0.66	3.52	1.00	0.62	1.57	**1.01**	**1.00**	**1.02**	0.97	0.26	3.60
Longueuil	Speeding’ light injuries	0.88	0.56	1.35	0.97	0.63	1.50	0.99	0.50	1.95	1.20	0.47	2.99
	Speeding’ KSI	0.83	0.38	1.75	0.74	0.32	1.68	0.78	0.22	2.69	0.97	0.31	3.05
Sherbrooke	Speeding’ light injuries	0.96	0.57	1.63	1.00	0.61	1.61	0.81	0.27	2.33	1.20	0.48	3.10
	Speeding’ KSI^†^	0.75	0.28	1.98	0.82	0.54	1.25

Models are adjusted for lags, time trends, daily temperature average, the Cannabis Act and sine and cosine pair of functions. The Autocorrelation and partial autocorrelation information, and Ljung-Box and Bartlett tests values for each model are availableonline supplemental figures S21–S30 and online supplemental table S9, respectively. Bold outcomes indicate p<0.05.

* The QCnPI was rescaled to facilitate the interpretation of the outcomes.

† Speeding KSI incidents were not reported during semesters 1 and 2.

IRR, incidence rate ratios; KSI, killed or severely injured; QCnPI, Québec Non-Pharmaceutical Interventions Index.

### Differences across semesters

Online supplemental tables S5–S6 present Z-scores and their corresponding p values. In most cases, the IRRs across semesters were not significantly different from each other at a p value threshold of <0.05. For alcohol-related light injuries, both Montréal and Québec showed significantly lower IRR values in semester 2 compared with semester 3. In Longueuil, a more modest result was observed, with the IRR in semester 1 lower than in semester 2 (p=0.07). In Laval, the IRR in semester 3 was lower than in semester 4 (p=0.09). For speeding-related light injuries, only Montréal showed notable differences across semesters, with semester 1 having lower values compared with semesters 3 and 4 and semester 2 showing lower values compared with semester 4. Finally, a modest result was observed in Laval, where semester 2 had a lower IRR compared with semester 4 (p=0.07).

## Discussion

This study offers a detailed examination of how COVID-19 NPIs influenced road safety, specifically analysing alcohol-related and speeding-related traffic injuries and fatalities (KSI) across five major cities in Québec: Montréal, Québec city, Laval, Longueuil and Sherbrooke. The use of the QCnPI-Index enabled a systematic assessment of changes in road safety outcomes across different pandemic phases.

Our findings indicate that the QCnPI-Index was associated with significant reductions in alcohol-related light injuries in Montréal, Québec city and Longueuil during the early pandemic phases (semesters 1 and 2). This reduction aligns with decreased traffic due to NPIs like curfews and business closures, particularly of bars and restaurants, which had restrictions on alcohol sales after midnight. These NPIs likely reduced opportunities for alcohol consumption, subsequently lowering alcohol-related traffic incidents. However, this decrease in injuries did not correspond with a significant reduction in alcohol-related fatalities (KSI), which remained relatively stable across cities.

In semester 3, alcohol-related injuries increased in most cities as QCnPI-Index values declined, suggesting a possible rebound effect as restrictions eased. Sherbrooke’s unique pattern, with increases persisting into semester 4, underscores the importance of localised analyses to fully understand pandemic measures’ impacts.

For speeding-related incidents, results were less consistent. The QCnPI-Index showed no strong overall association with speeding-related injuries or fatalities across most cities. This may indicate that, while reduced traffic volumes allowed for higher speeds,^30^ NPIs did not uniformly affect speeding-related outcomes. Notably, Montréal and Laval saw increases in speeding-related KSI and light injuries during semester 3, corresponding with the relaxation of some NPIs. These increases may reflect changes in driver behaviour as restrictions lifted, with drivers resuming more typical patterns and perceiving enforcement as less stringent.^31^

The varying impacts across cities emphasise the complexity of NPIs’ effects on road safety. Cities with initially higher rates of alcohol-related injuries, like Longueuil, experienced a more pronounced effect from NPIs, whereas cities like Laval and Sherbrooke exhibited unique trends. This localised response to NPIs, alongside specific city characteristics, highlights the importance of tailored public health and traffic safety interventions.

This study has several limitations. First, we did not consider factors such as age or sex, which are crucial for understanding speeding and alcohol-related incidents variation. Second, although we analysed data across four distinct semesters to capture temporal differences, we did not account for short-term or long-term effects within each semester. Third, the QCnPI-Index’s varied impacts might have changed traffic patterns from the usual two-peak pattern (morning and afternoon) to a single afternoon peak in some cities. Our analysis focused on daily variations rather than hourly variations, which might explain these peak changes. Fourth, the QCnPI-Index might have interacted with specific road safety measures during COVID-19, potentially affecting its influence on speeding and alcohol-related outcomes. We could not account for instance for specific road safety measures implemented during the pandemic, such as changes in police enforcement of speed limits. Lastly, while the SAAQ systematises crash data, there could be inaccuracies in police reports, potentially leading to misclassification of severe and minor injuries.

## Conclusion

In summary, the study demonstrates that while NPIs were significantly associated with a reduction in reducing alcohol-related light injuries in several cities, particularly during the first two semesters, the effects on speeding-related incidents were less uniform. The complex interplay between pandemic restrictions, behavioural changes and road safety underscores the need for continuous monitoring and adjustment of public health interventions to balance traffic safety with broader public health objectives. The insights gained from this analysis are crucial for informing future pandemic response strategies and enhancing road safety management during emergencies.

## Supplementary material

10.1136/ip-2024-045481 online supplemental file 1

## Data Availability

Data may be obtained from a third party and are not publicly available.

## References

[1] Nazif-Muñoz JI, Peña S, Oulhote Y (2021). The global viralization of policies to contain the spreading of the COVID-19 pandemic: Analyses of school closures and first reported cases. PLoS One.

[2] Santamaria C, Sermi F, Spyratos S (2020). Measuring the impact of COVID-19 confinement measures on human mobility using mobile positioning data. A European regional analysis. Saf Sci.

[3] Timme S, Cook B, Schipfer M (2022). Forced to stay at home—impact of curfews on mood during a pandemic for individuals with exercise dependence. *Ger J Exerc Sport Res*.

[4] Primc K, Slabe-Erker R The Success of Public Health Measures in Europe during the COVID-19 Pandemic. Sustainability.

[5] Yasin YJ, Grivna M, Abu-Zidan FM (2021). Global impact of COVID-19 pandemic on road traffic collisions. World J Emerg Surg.

[6] McCoy LG, Smith J, Anchuri K (2020). Characterizing early Canadian federal, provincial, territorial and municipal nonpharmaceutical interventions in response to COVID-19: a descriptive analysis. CMAJ Open.

[7] McSween-Cadieux E, Lane J, Hong QN (2024). Production and use of rapid responses during the COVID-19 pandemic in Quebec (Canada): perspectives from evidence synthesis producers and decision makers. Health Res Policy Syst.

[8] Mamri A, Pereira CCM, Najafi Moghaddam Gilani V (2024). Towards a comprehensive COVID-19 non-pharmaceutical interventions’ index for the province of Québec. BMC Res Notes.

[9] Enam A, Rahman SM, Mahmud SMS (2023). Impacts of COVID-19-Related Non-Pharmaceutical Interventions on Mobility and Accidents in Bangladesh. Transp Res Rec.

[10] Katrakazas C, Michelaraki E, Sekadakis M (2020). A descriptive analysis of the effect of the COVID-19 pandemic on driving behavior and road safety. Transp Res Interdiscip Perspect.

[11] Nazif-Munoz BB, Brown TG (2024). The impact of non-pharmaceutical COVID-19 interventions on collisions, traffic injuries and fatalities across Québec. Transp Res Interdiscip Perspect.

[12] Tucker A, Marsh KL (2021). Speeding through the pandemic: Perceptual and psychological factors associated with speeding during the COVID-19 stay-at-home period. Accid Anal Prev.

[13] Hostiuc S, Radu D, Seretean L (2021). Driving under the influence of alcohol during the COVID-19 pandemic. Forensic Sci Int.

[14] van Hoving DJ, van Koningsbruggen C, de Man M (2021). Temporal changes in trauma according to alcohol sale restrictions during the South African national COVID-19 lockdown. Afr J Emerg Med.

[15] SAAQ (2023). Bilan routier 2021: baisse du nombre de personnes accidentées de 18,0 % par rapport à la moyenne de 2016 à 2020. https://saaq.gouv.qc.ca/saaq/documentation/bilan-routier.

[16] Québec I de la statistique du Population et composantes de l’accroissement démographique, par trimestre, québec, 2001-2024. https://statistique.quebec.ca/fr/produit/tableau/population-composantes-accroissement-demographique-trimestre-quebec.

[17] Government of Canada (2023). Historical data - climate - environment and climate change canada. https://climate.weather.gc.ca/historical_data/search_historic_data_e.html.

[18] Government of Canada (2023). Consolidated federal laws of canada, cannabis act. https://laws-lois.justice.gc.ca/eng/acts/c-24.5/.

[19] Nazif‐Munoz JI, Domínguez‐Cancino KA, Ouimet MC (2023). Did the cannabis recreational use law affect traffic crash outcomes in Toronto? Building evidence for the adequate number of authorised cannabis stores’ thresholds. Drug Alcohol Rev.

[20] Johansson P (1996). Speed limitation and motorway casualties: a time series count data regression approach. Accid Anal Prev.

[21] Theofilatos A, Yannis G (2014). A review of the effect of traffic and weather characteristics on road safety. Accident Analysis & Prevention.

[22] Cheng W, Gill GS, Choi S (2018). Comparative evaluation of temporal correlation treatment in crash frequency modelling. Transp A: Transp Sci.

[23] Quddus MA (2008). Time series count data models: An empirical application to traffic accidents. Accident Analysis & Prevention.

[24] Hay RA, McCleary R (1980). Applied Time Series Analysis For The Social Sciences.

[25] Cox NJ (2006). Speaking Stata: In Praise of Trigonometric Predictors. Stata J.

[26] Hoffmann K, Pischon T, Schulz M (2008). A statistical test for the equality of differently adjusted incidence rate ratios. Am J Epidemiol.

[27] Linden A (2015). Conducting Interrupted Time-series Analysis for Single- and Multiple-group Comparisons. The Stata J Promoting comm on stats and Stata.

[28] Woodward WA, Gray HL, Elliott AC (2017). Applied Time Series Analysis with R.

[29] StataCcorp (2019). Statistical software:release 16.

[30] Kraemer MUG, Yang C-H, Gutierrez B (2020). The effect of human mobility and control measures on the COVID-19 epidemic in China. Science.

[31] Watson-Brown N, Nicolls M, Mardani A (2024). A qualitative study of the first COVID-19 pandemic lockdown: The impact on alcohol consumption and driving behaviours. Transp Res Part F: Traffic Psychol Behav.

