# Supplementary material for: Influence of non-pharmaceutical COVID-19 interventions on speed-related and alcohol-related traffic injuries in five cities of the province of Québec, Canada
Source: Inj Prev. 2025 Jan 2;32(5):e045481. doi: 10.1136/ip-2024-045481 (PMC13638290; doi:10.1136/ip-2024-045481)
Supplement: Supplementary file 1 [file ip-32-5-s001.pdf]

Supplementary Materials

Figures S1 to S5: Incidence Rate Ratios of Alcohol-related crashes and transformation-Indexes

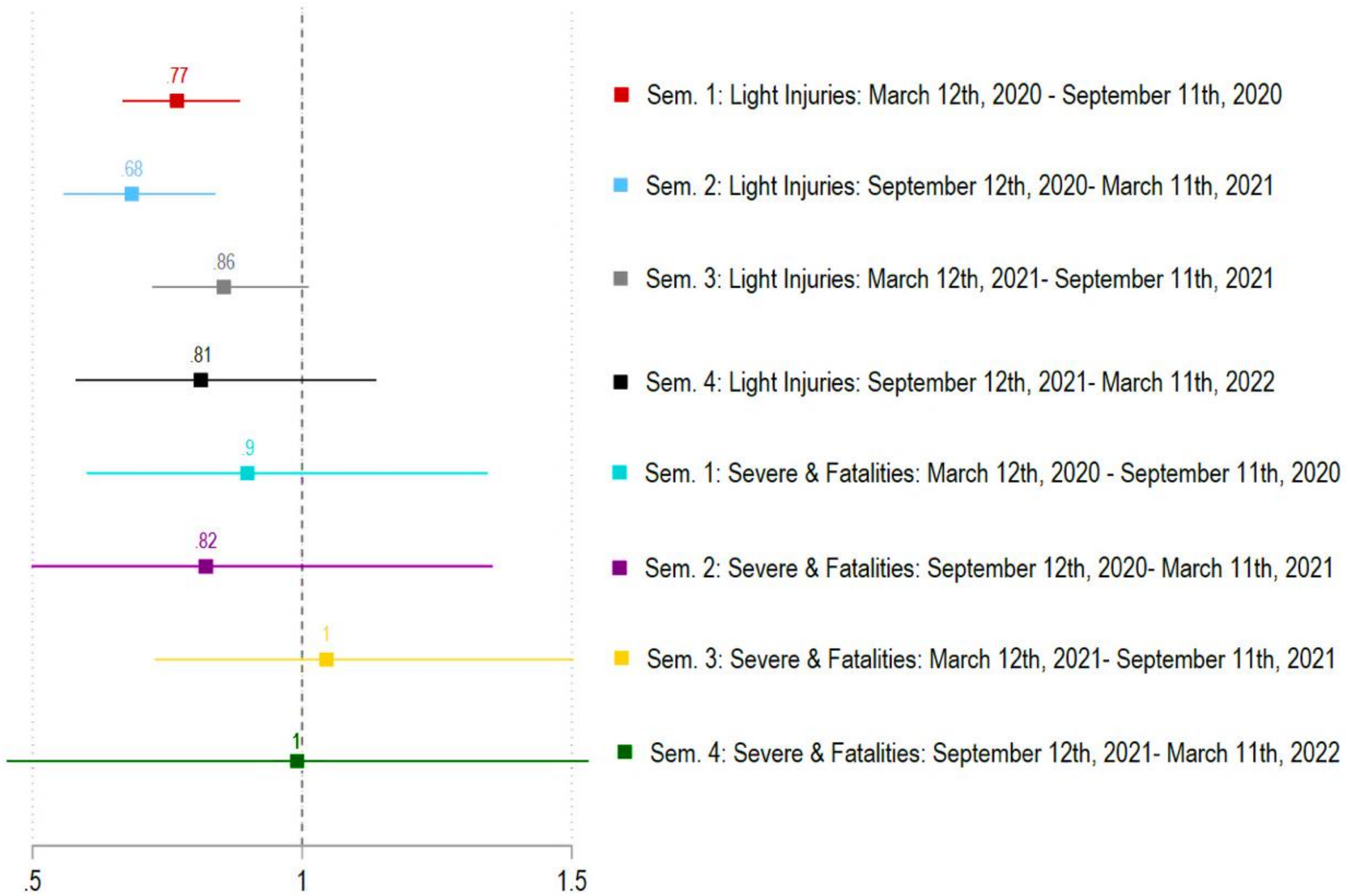

Figure S1. City of Montreal

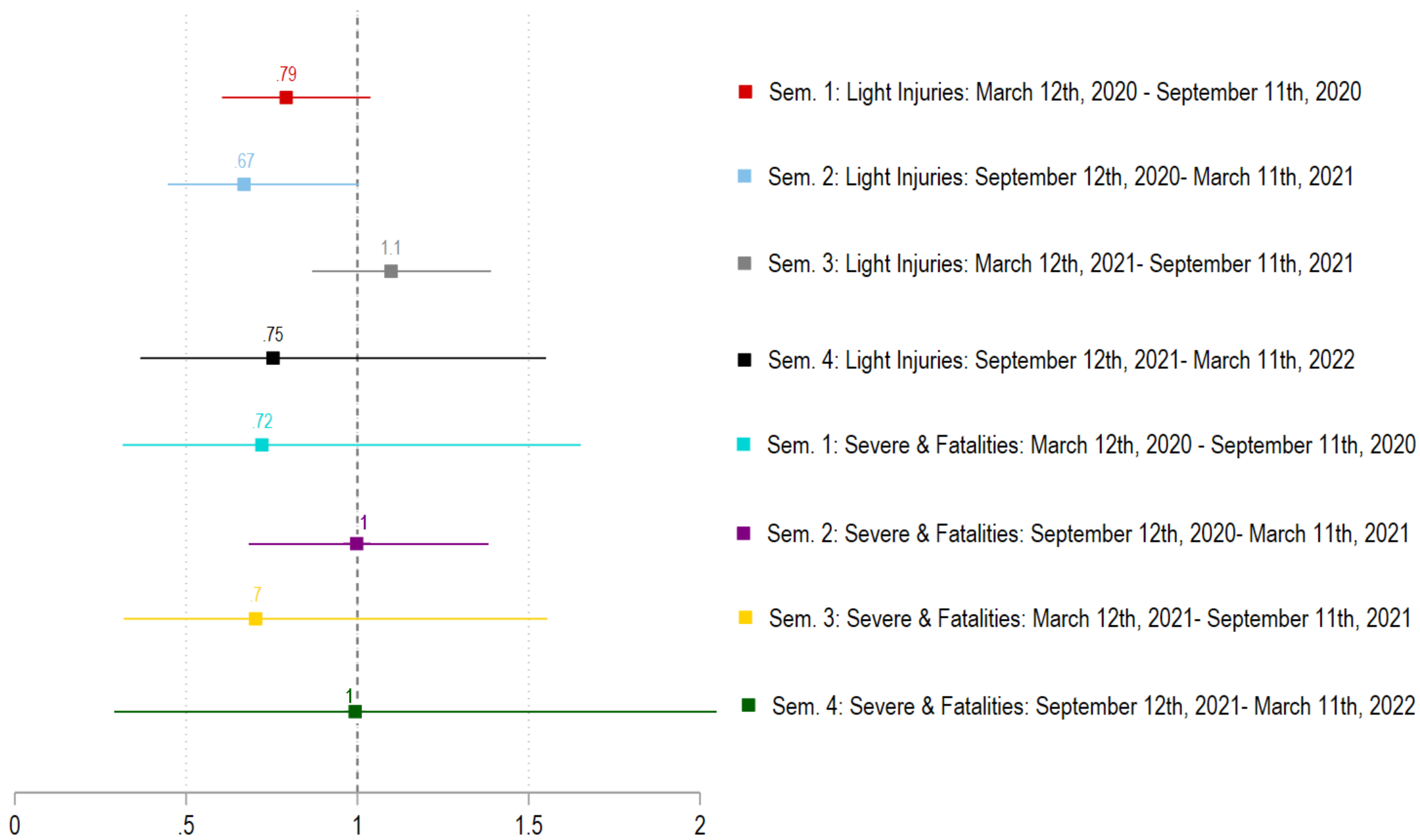

Figure S2. City of Quebec

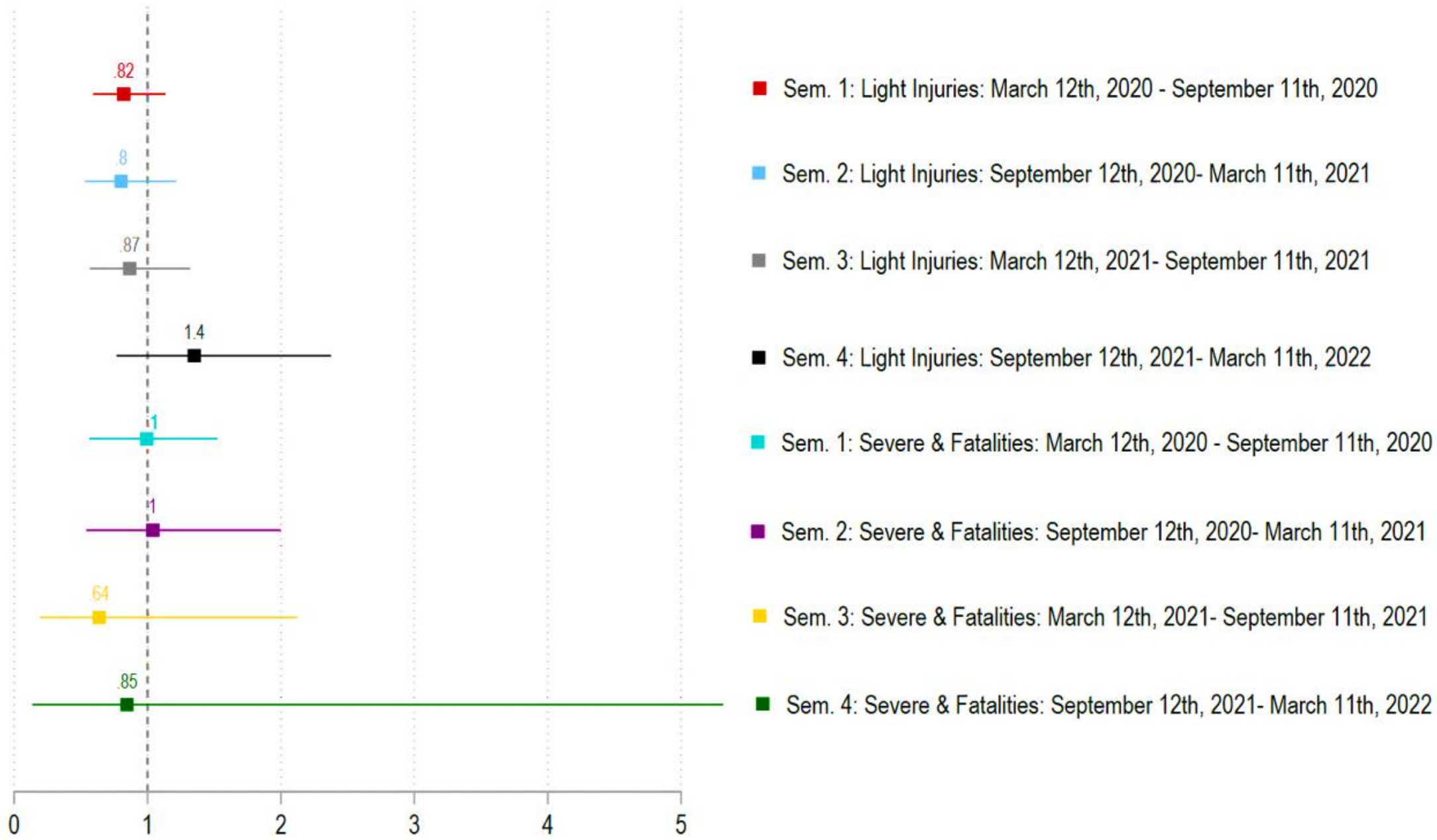

Figure S3. City of Laval

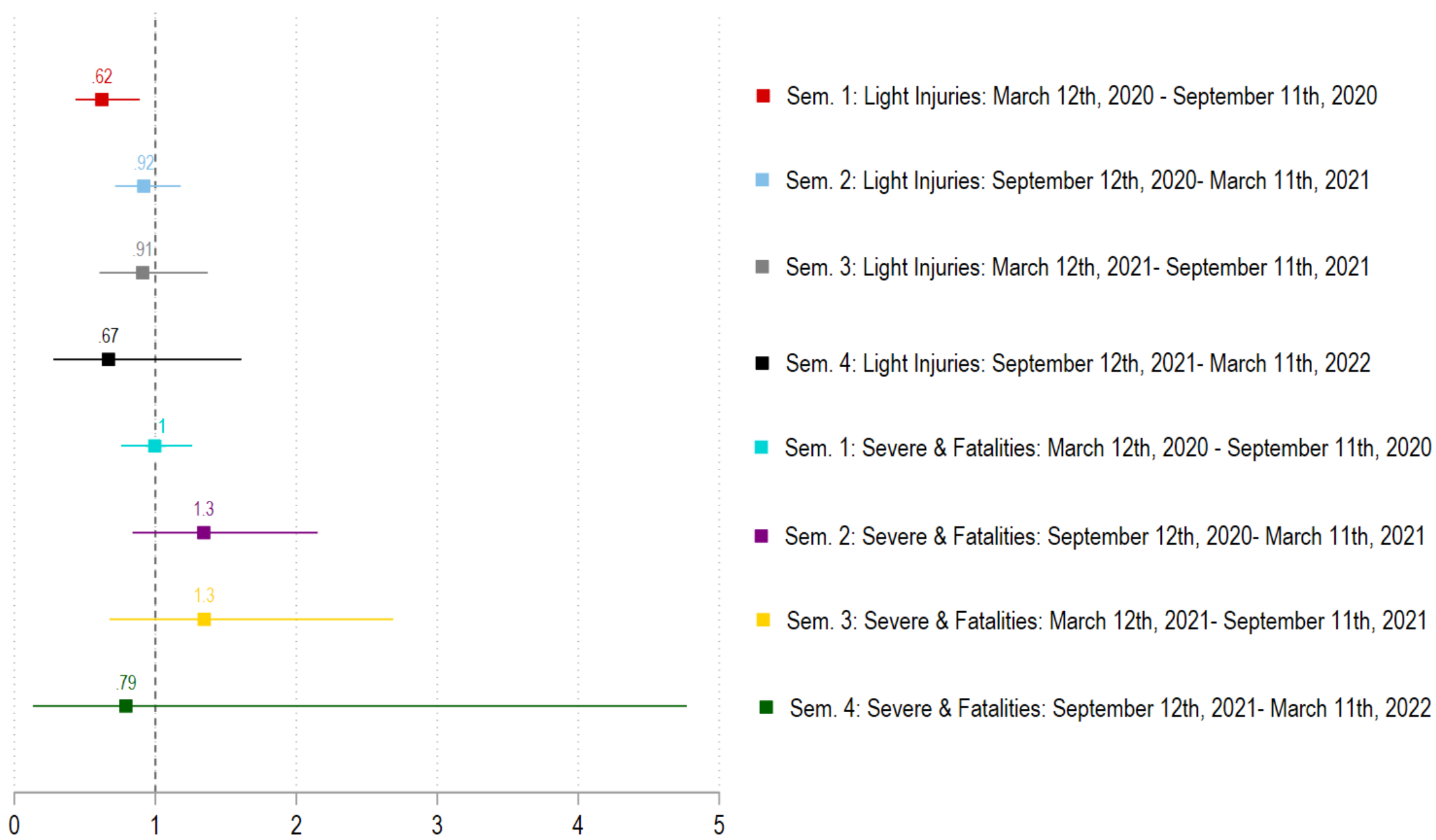

Figure S4. City of Longueuil

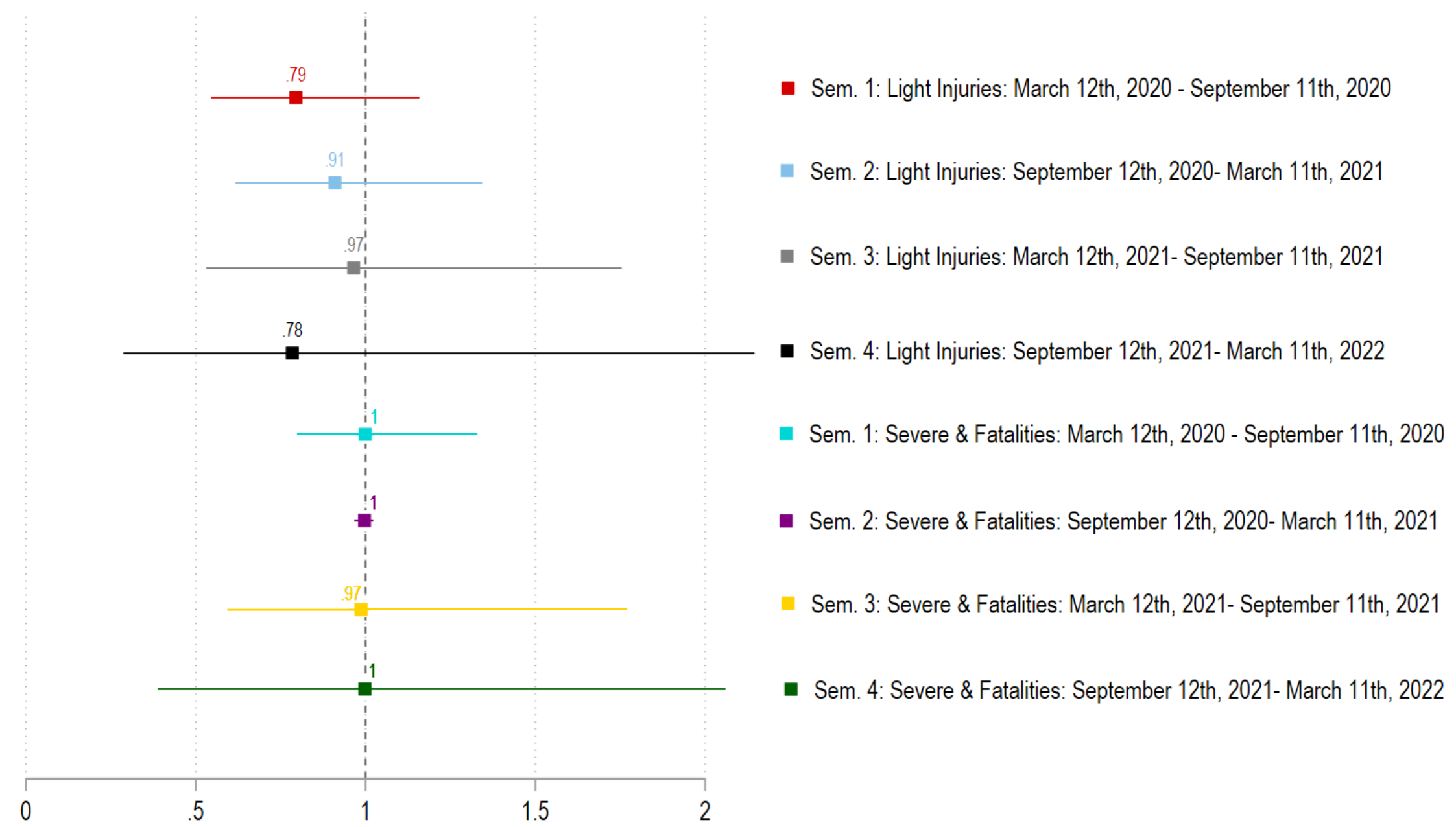

Figure S5. City of Sherbrooke

Figures S6 to 10: Incidence Rate Ratios of Speeding-related crashes and transformation-Indexes

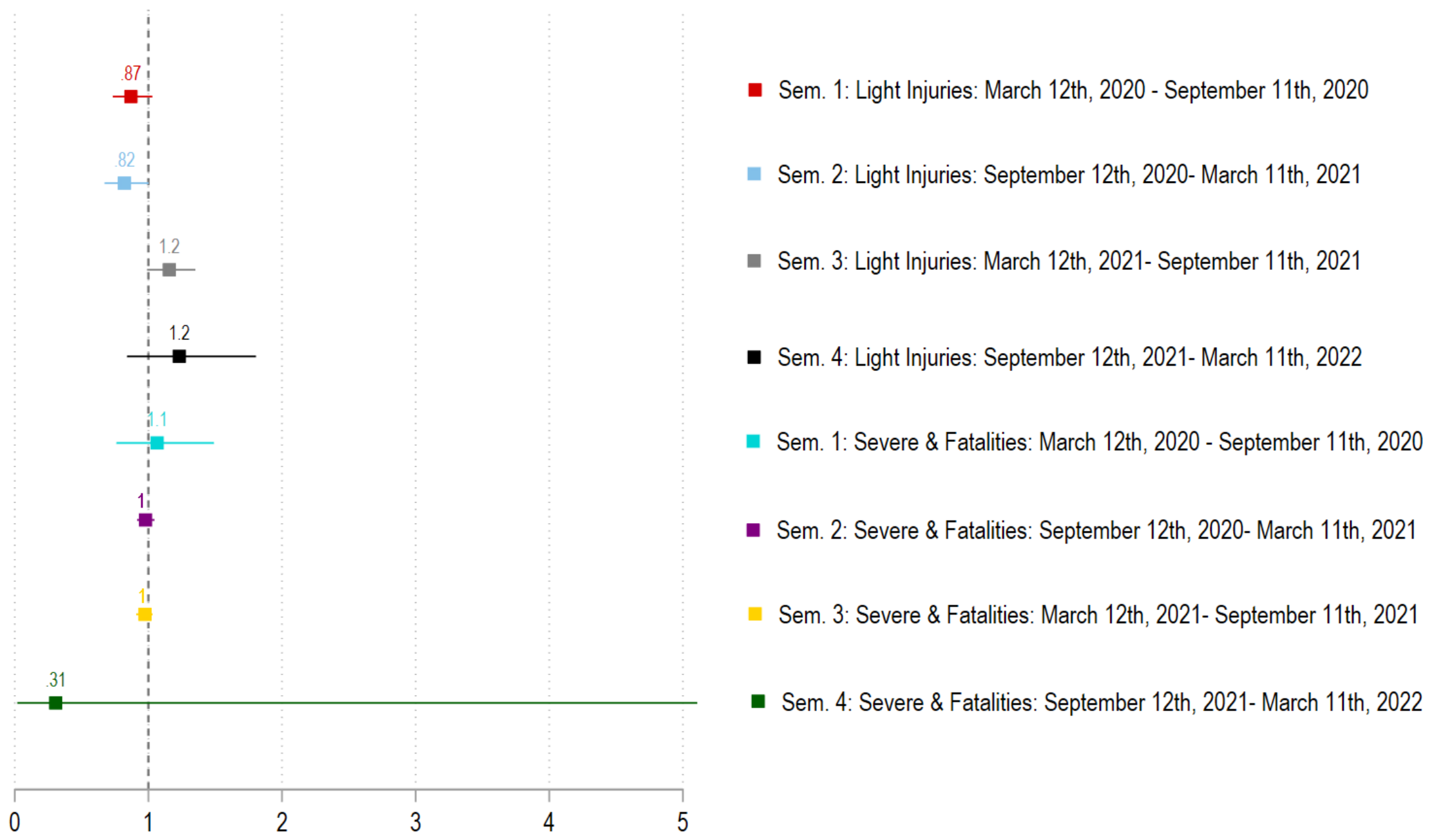

Figure S6. City of Montreal

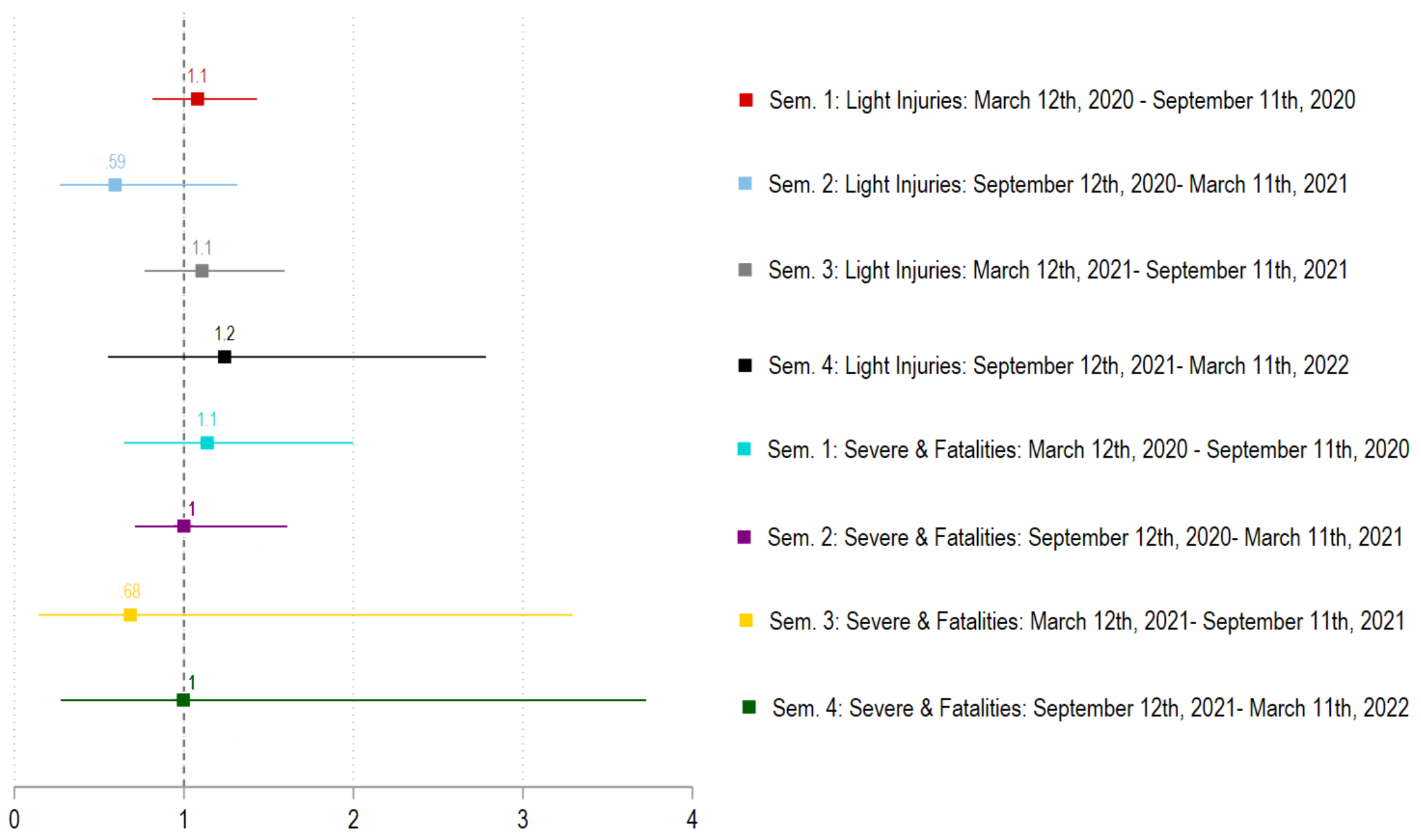

Figure S7. City of Quebec

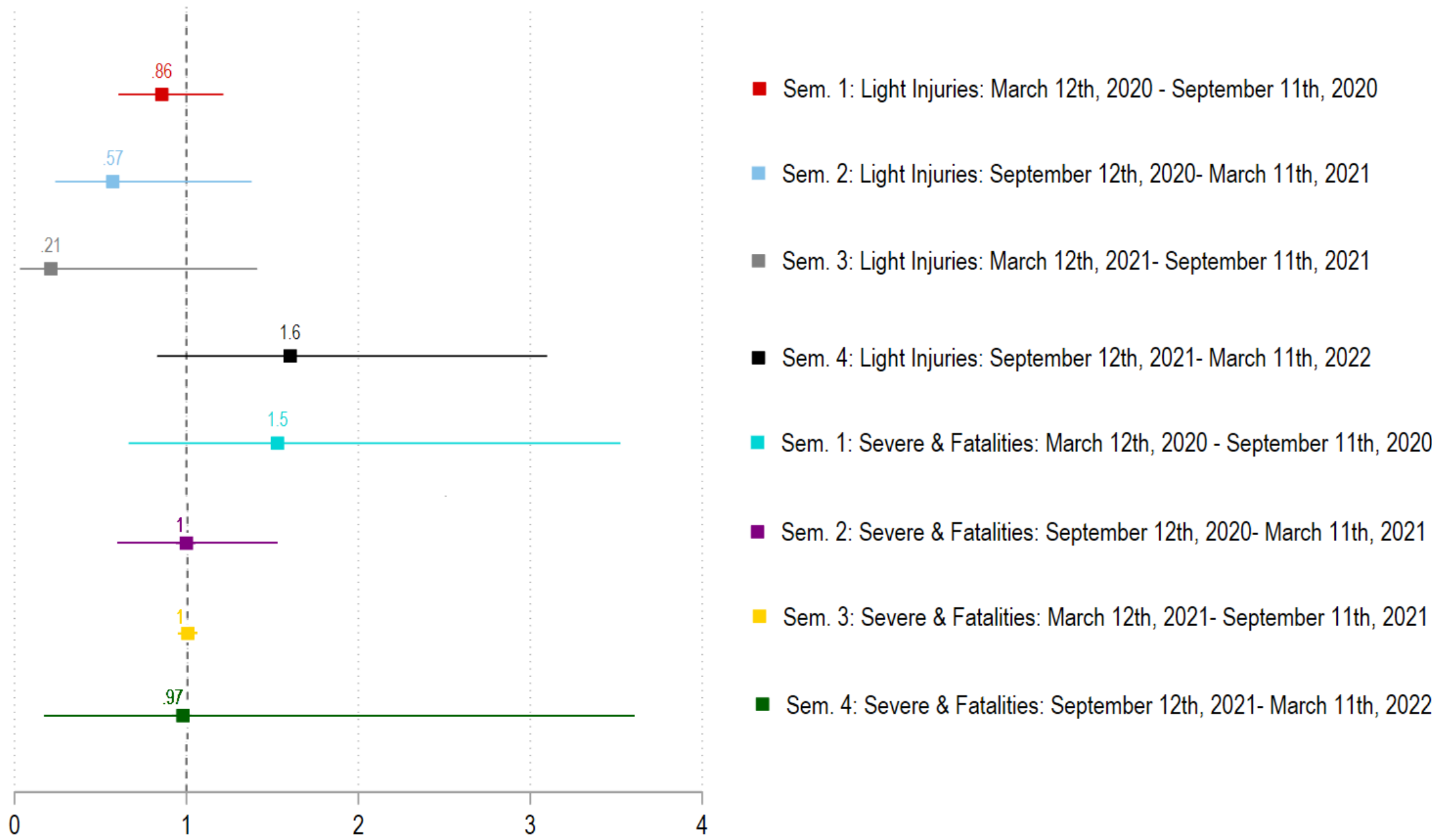

Figure S8. City of Laval

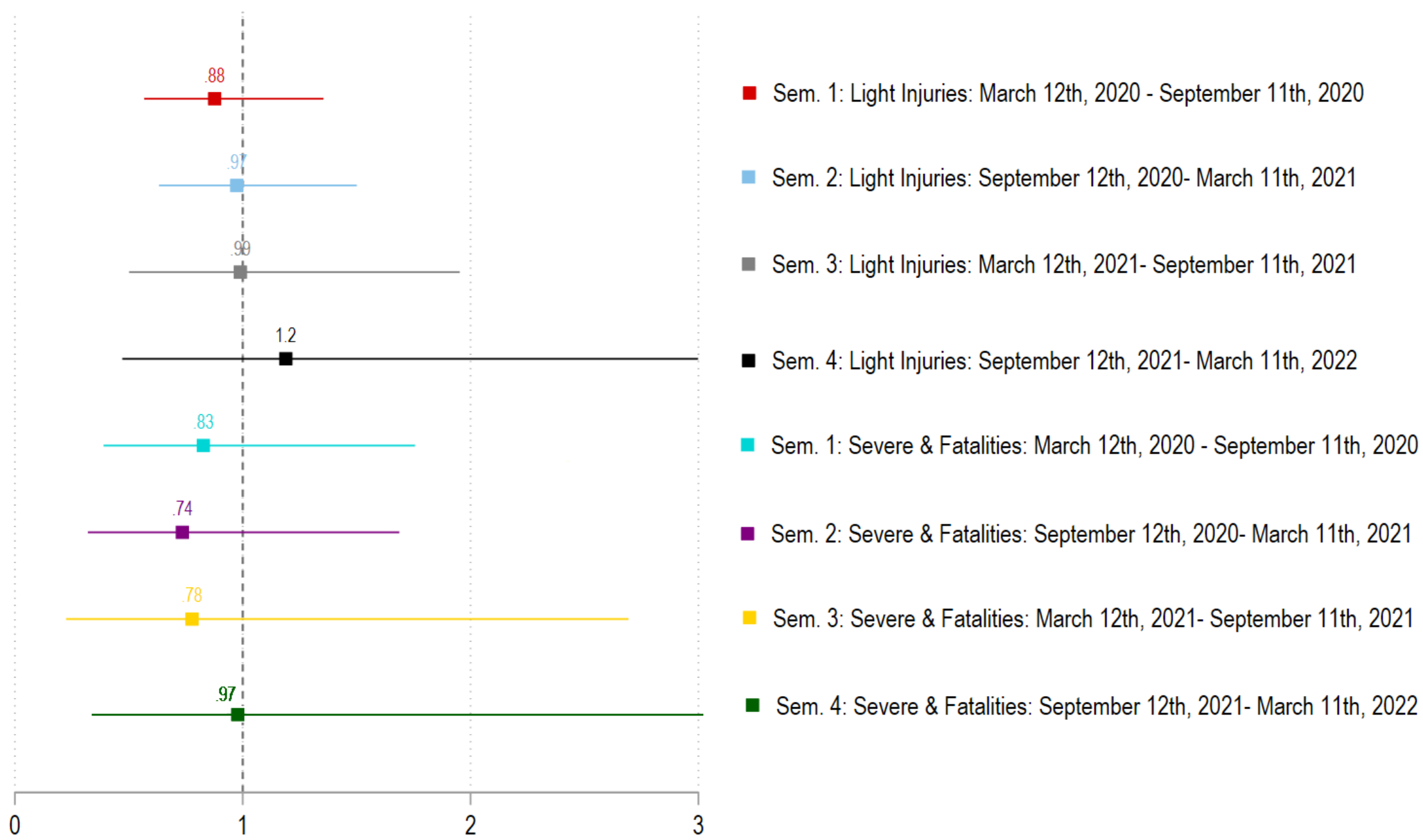

Figure S9. City of Longueuil

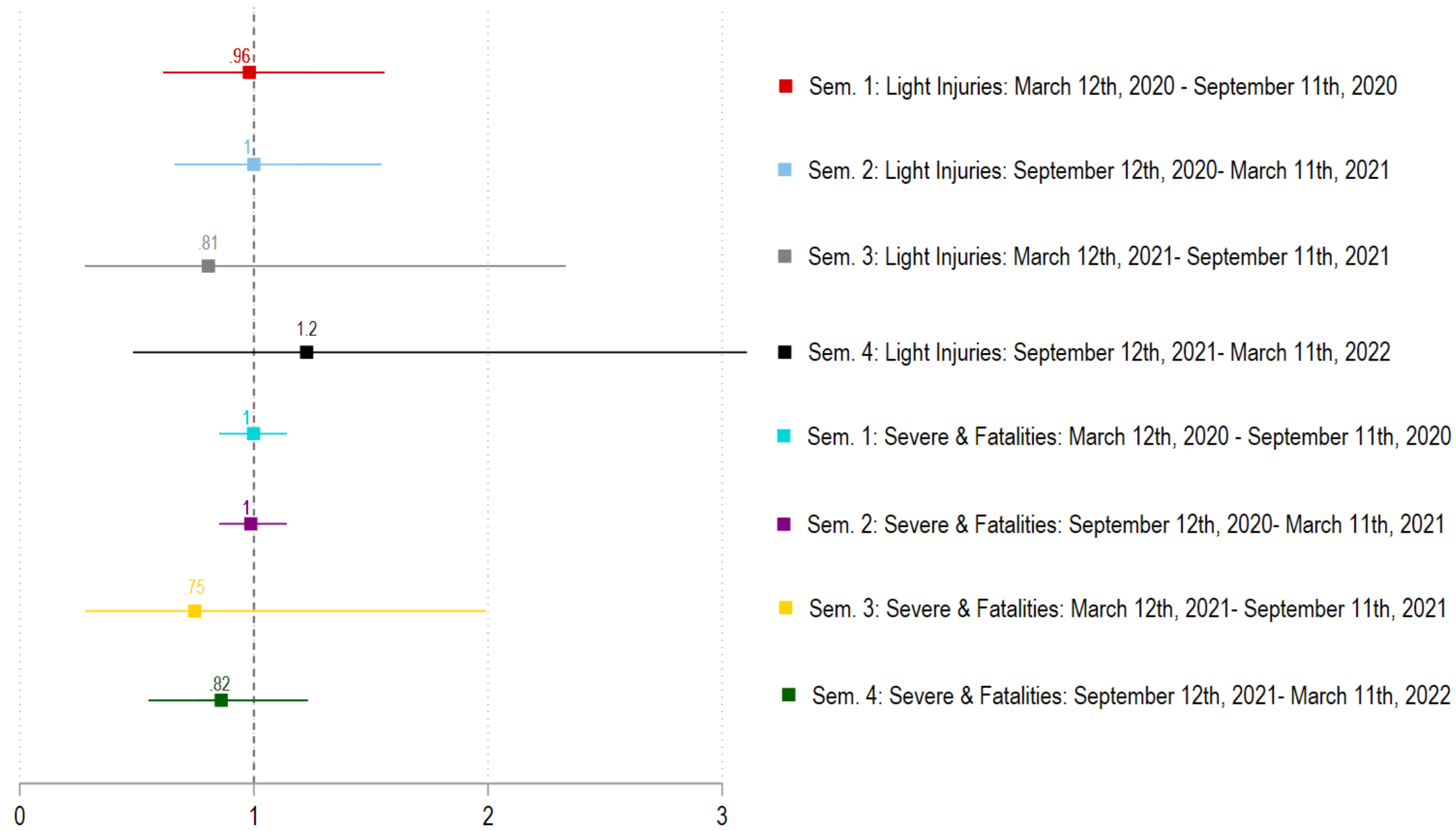

Figure S10. City of Sherbrooke

Figures S11-S20 Autocorrelation (AC) and Partial Auto Correlations (PAC) of residuals of Alcohol-related crashes without interventions.

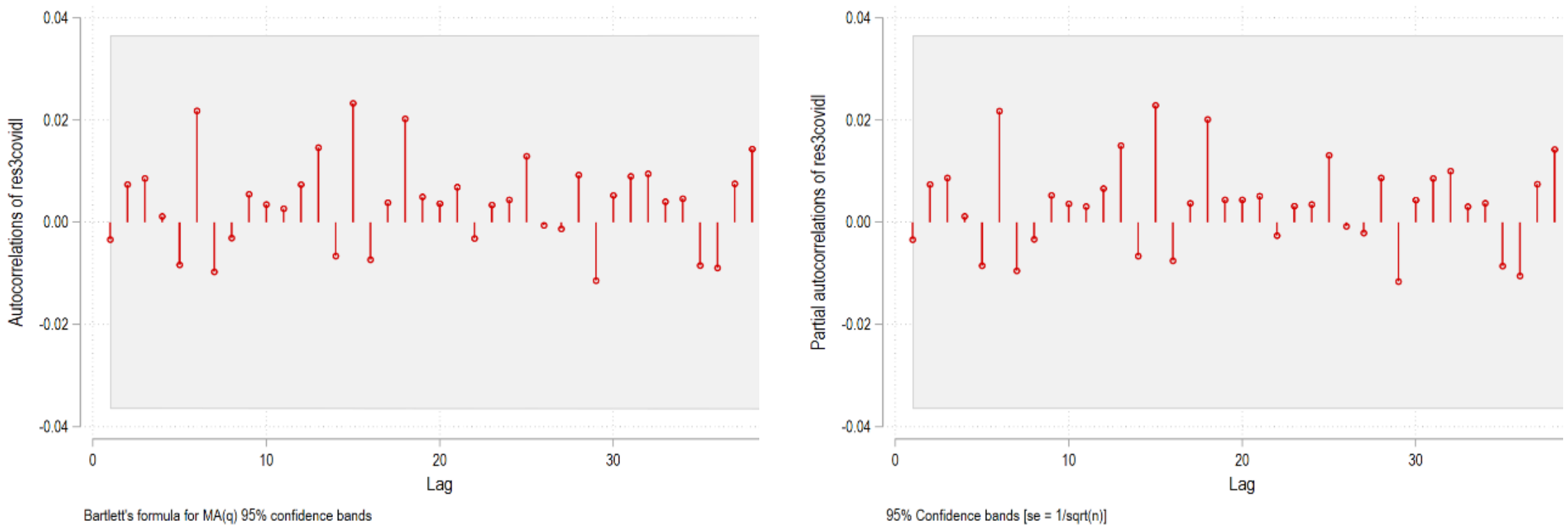

a b

Figure S11. Light Injuries (Alcohol): a: AC, b: PAC, City of Montreal

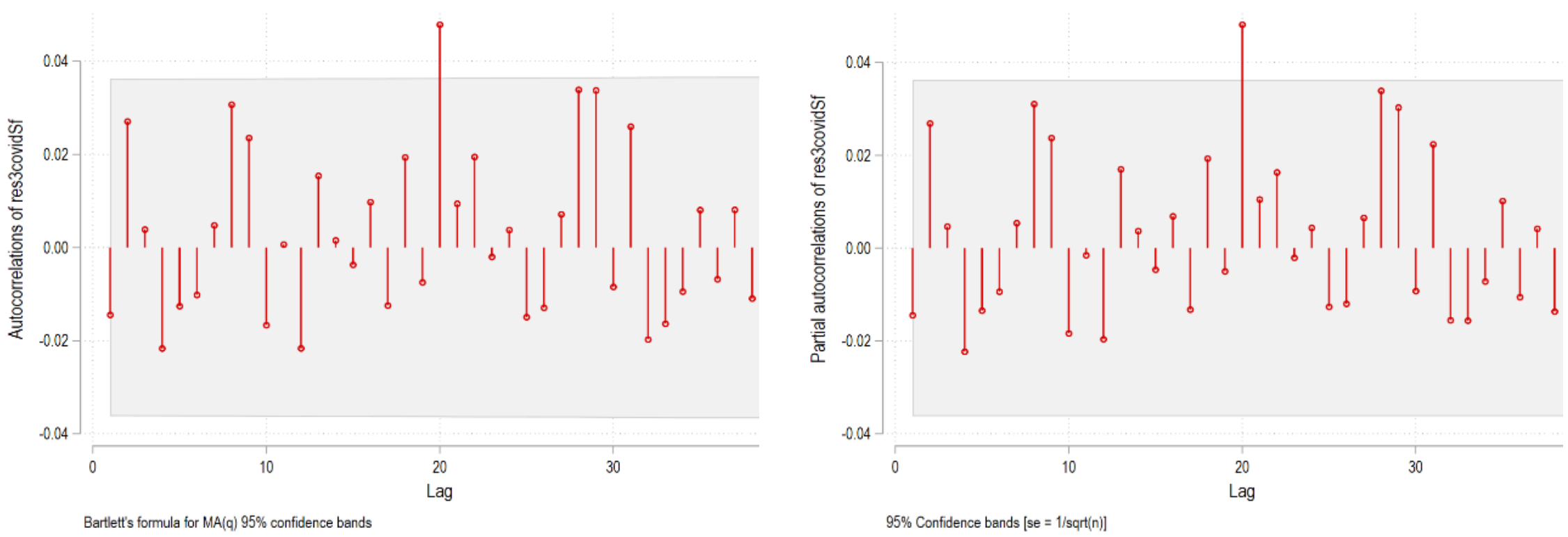

a b

Figure S12. Severe Injuries-Traffic Fatality (Alcohol): a: AC, b: PAC, City of Montreal

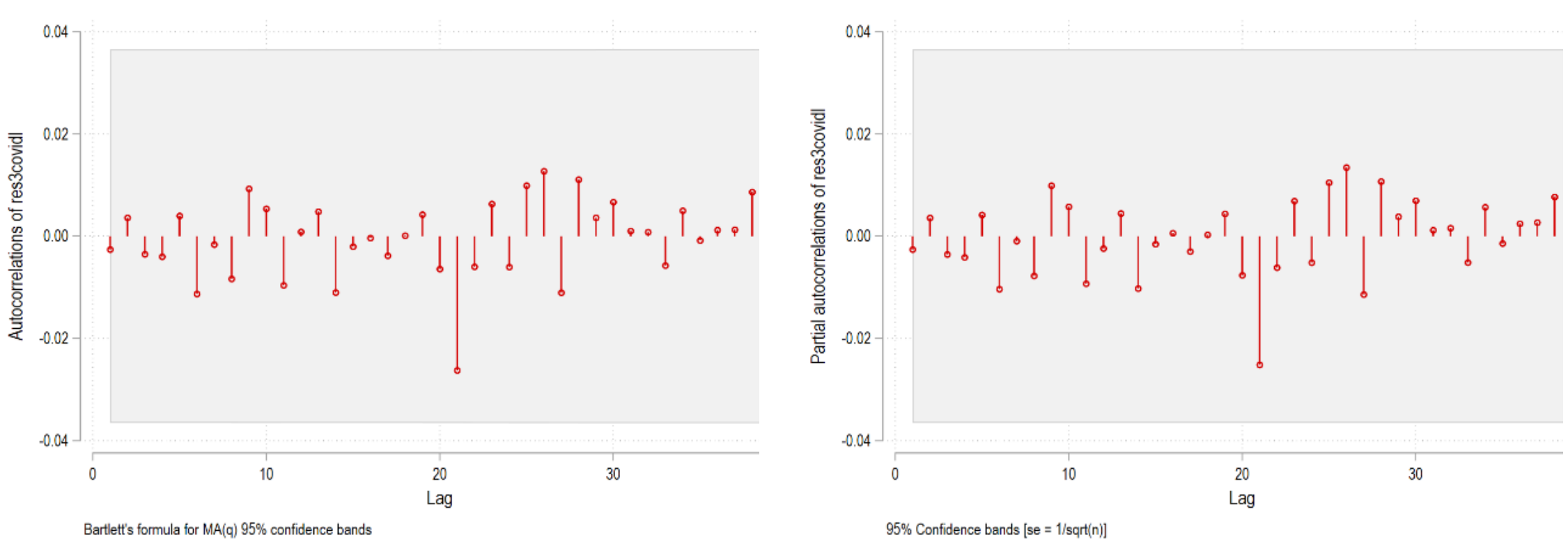

a b

Figure S13. Light Injuries (Alcohol): a: AC, b: PAC, City of Quebec

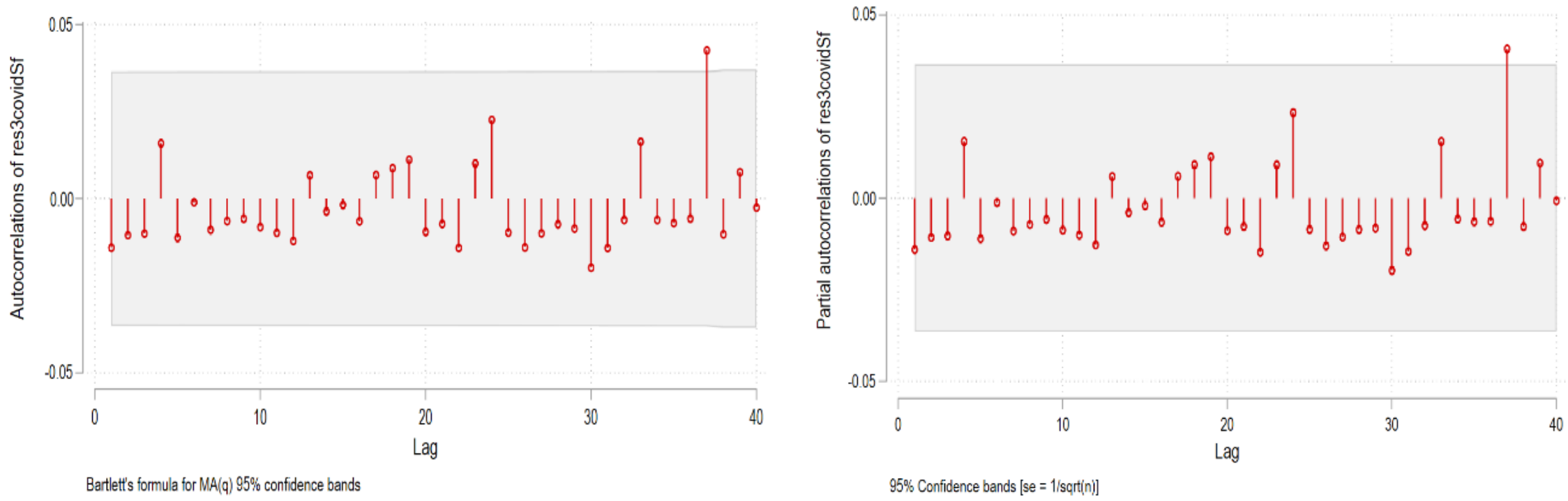

a b

Figure S14. Severe Injuries-Traffic Fatality (Alcohol): a: AC, b: PAC, City of Quebec

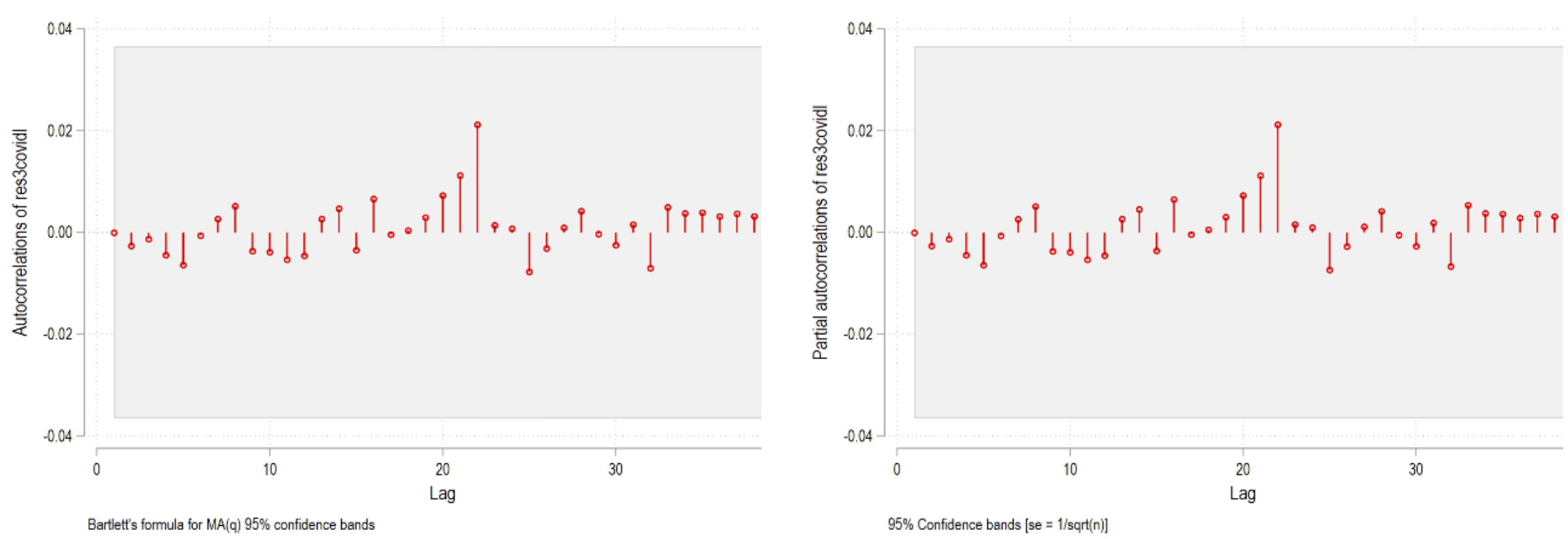

a b

Figure S15. Light Injuries (Alcohol): a: AC, b: PAC, City of Laval

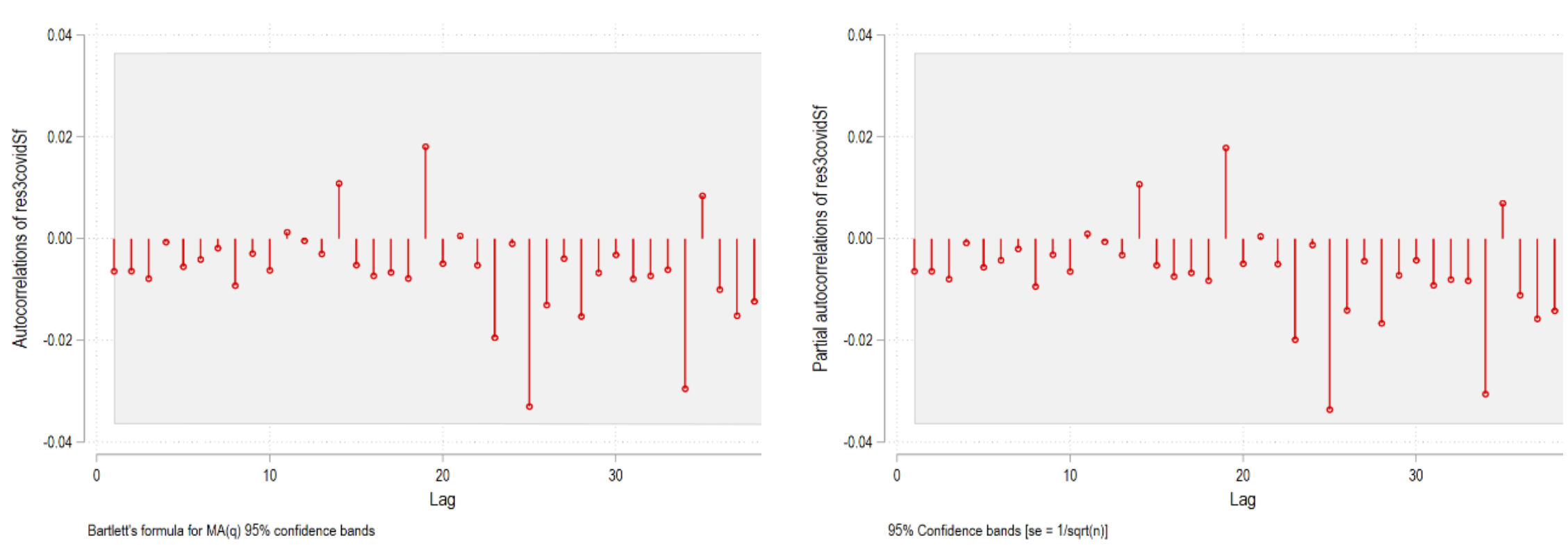

a b

Figure S16. Severe Injuries-Traffic Fatality (Alcohol): a: AC, b: PAC, City of Laval

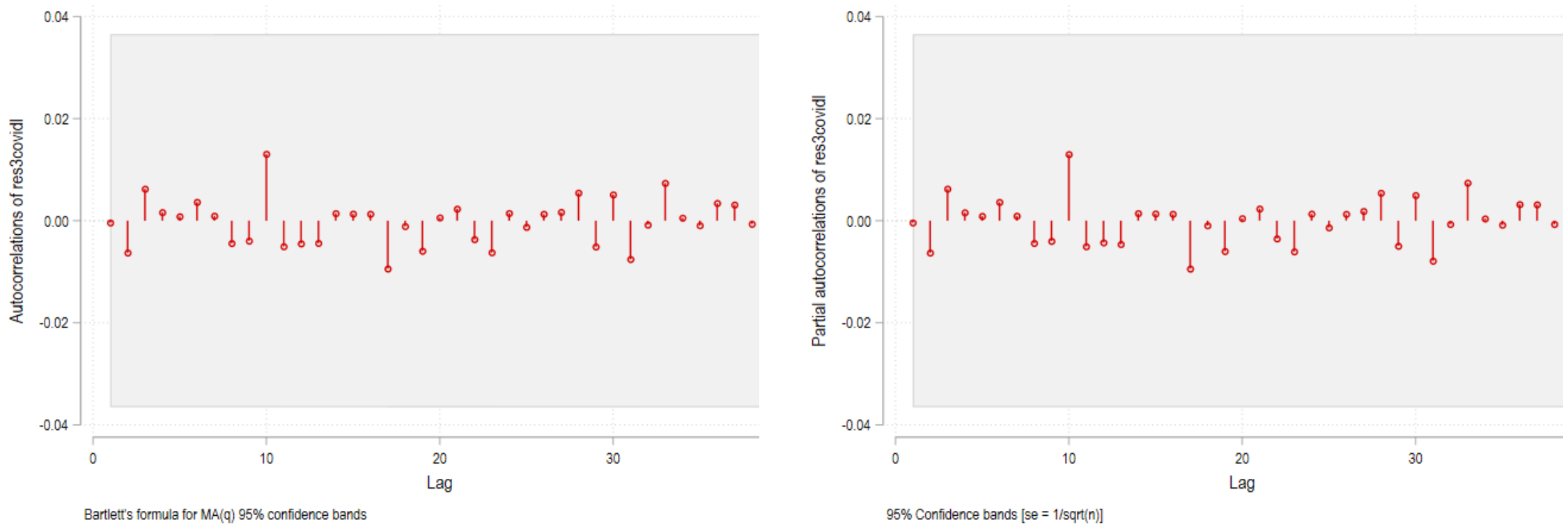

a b

Figure S17. Light Injuries (Alcohol): a: AC, b: PAC, City of Longueuil

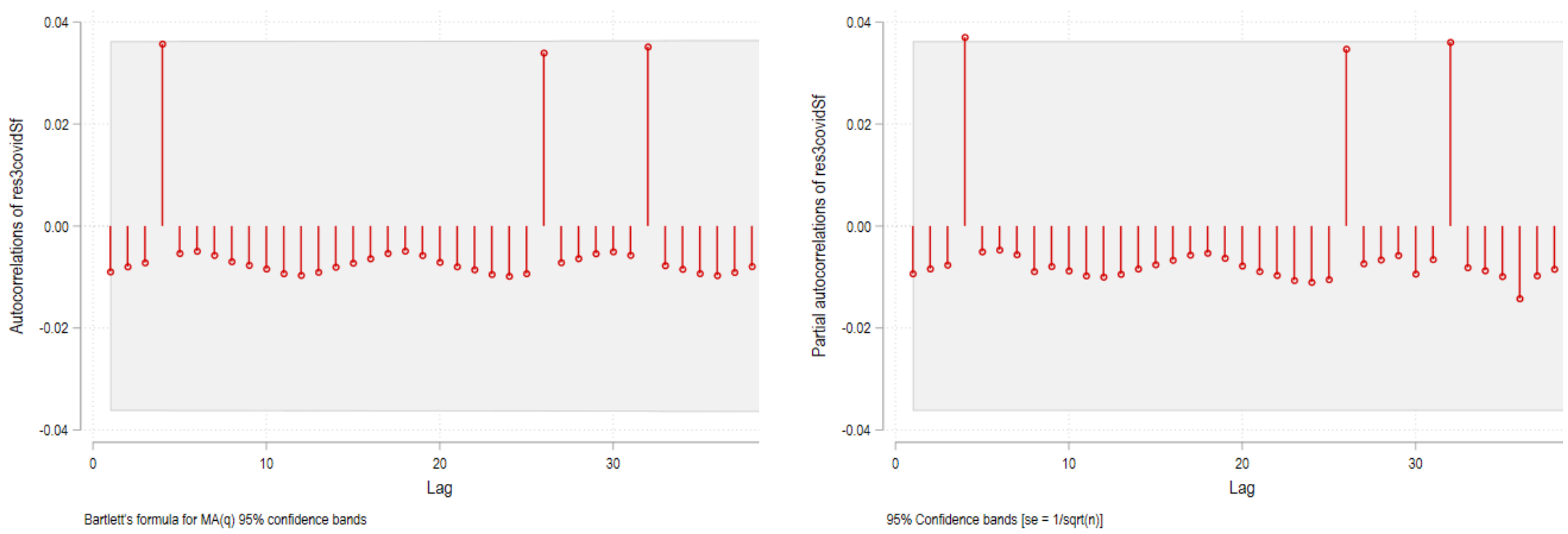

a b

Figure S18. Severe Injuries-Traffic Fatality (Alcohol): a: AC, b: PAC, City of Longueuil

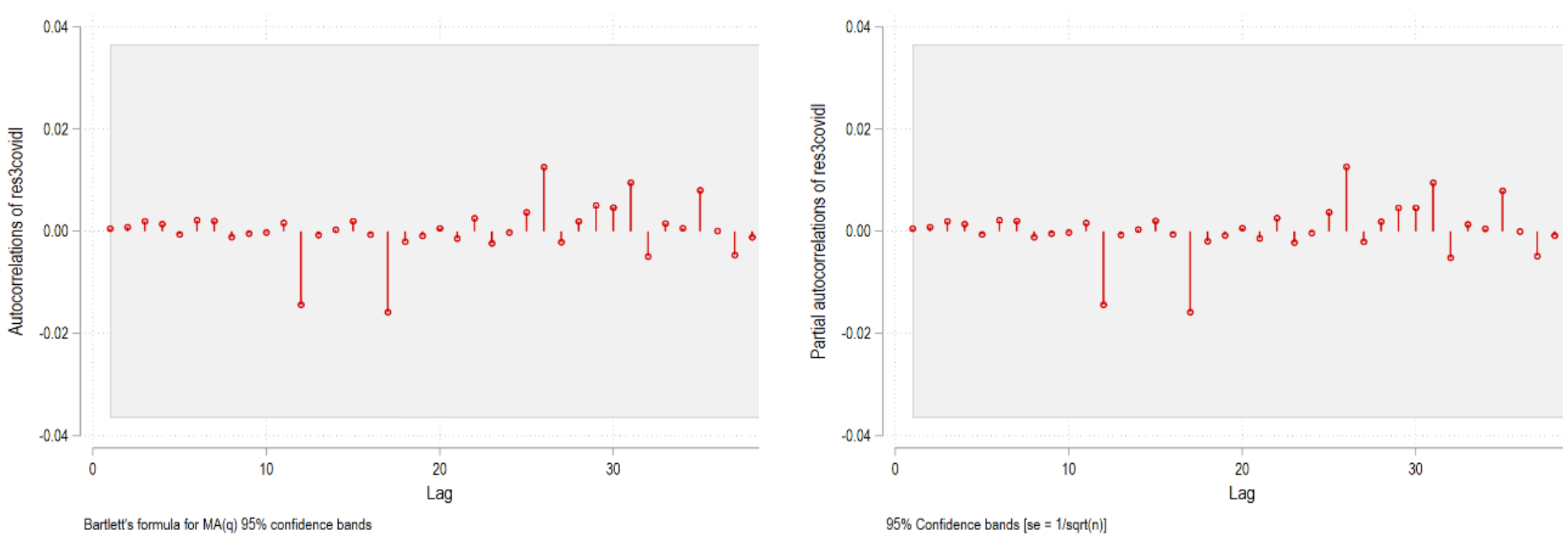

a b

Figure S19. Light Injuries (Alcohol): a: AC, b: PAC, City of Sherbrooke

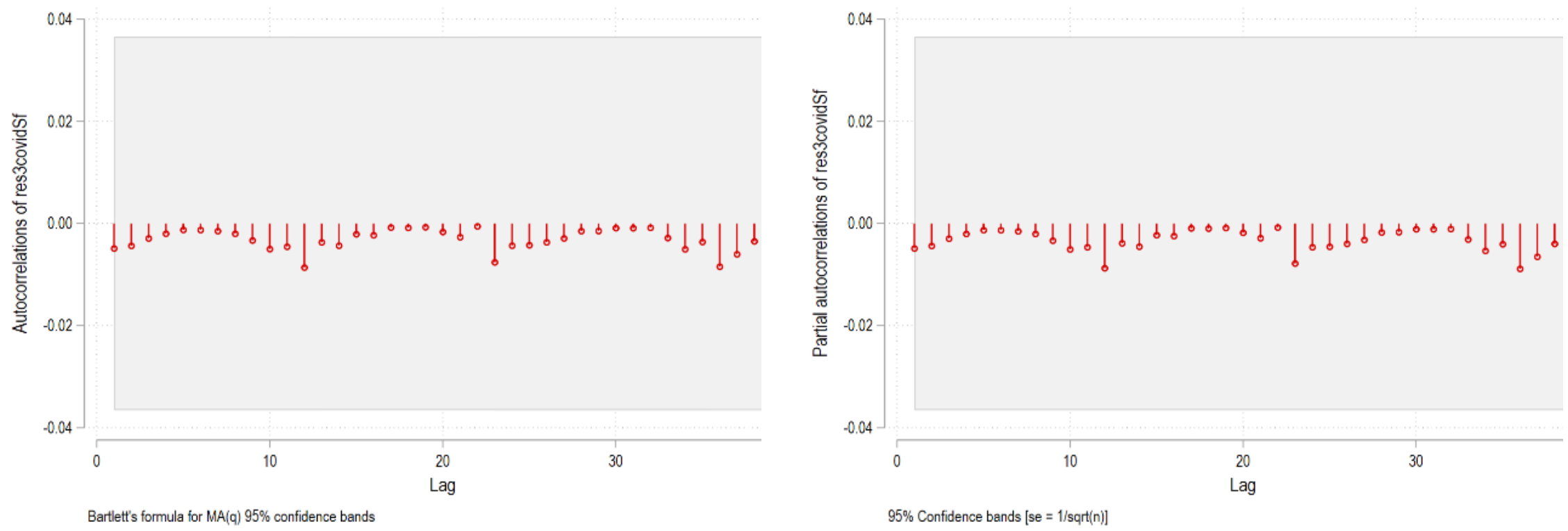

a b

Figure S20. Severe Injuries-Traffic Fatality (Alcohol): a: AC, b: PAC, City of Sherbrooke

Figures S21-S30. Autocorrelation (AC) and Partial Auto Correlations (PAC) of residuals of Speeding-related crashes without interventions

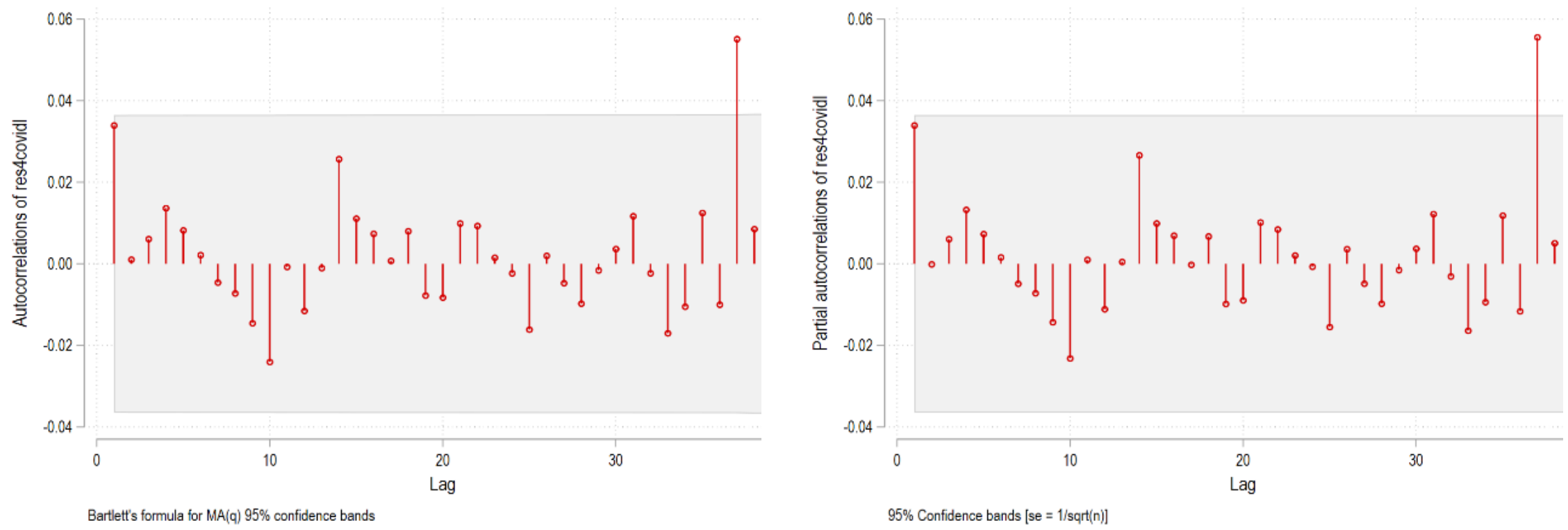

a b

Figure S21. Light Injuries (Speeding): a: AC, b: PAC, City of Montreal

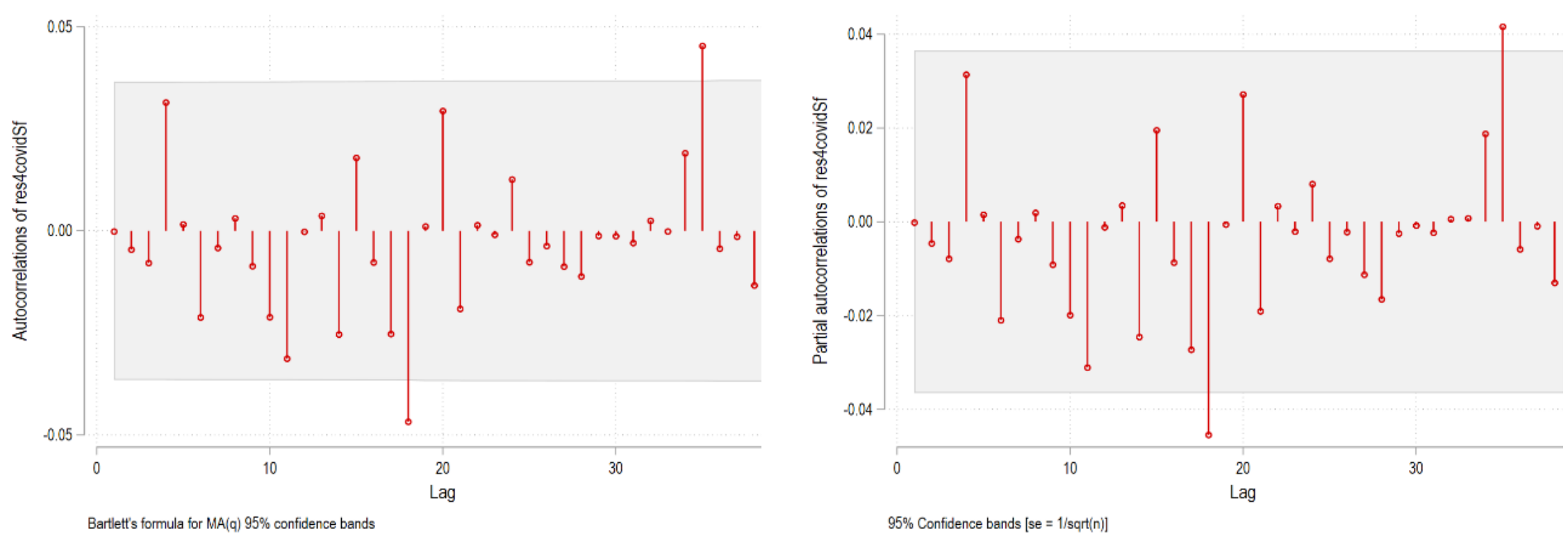

a b

Figure S22. Severe Injuries-Traffic Fatality (Speeding): a: AC, b: PAC, City of Montreal

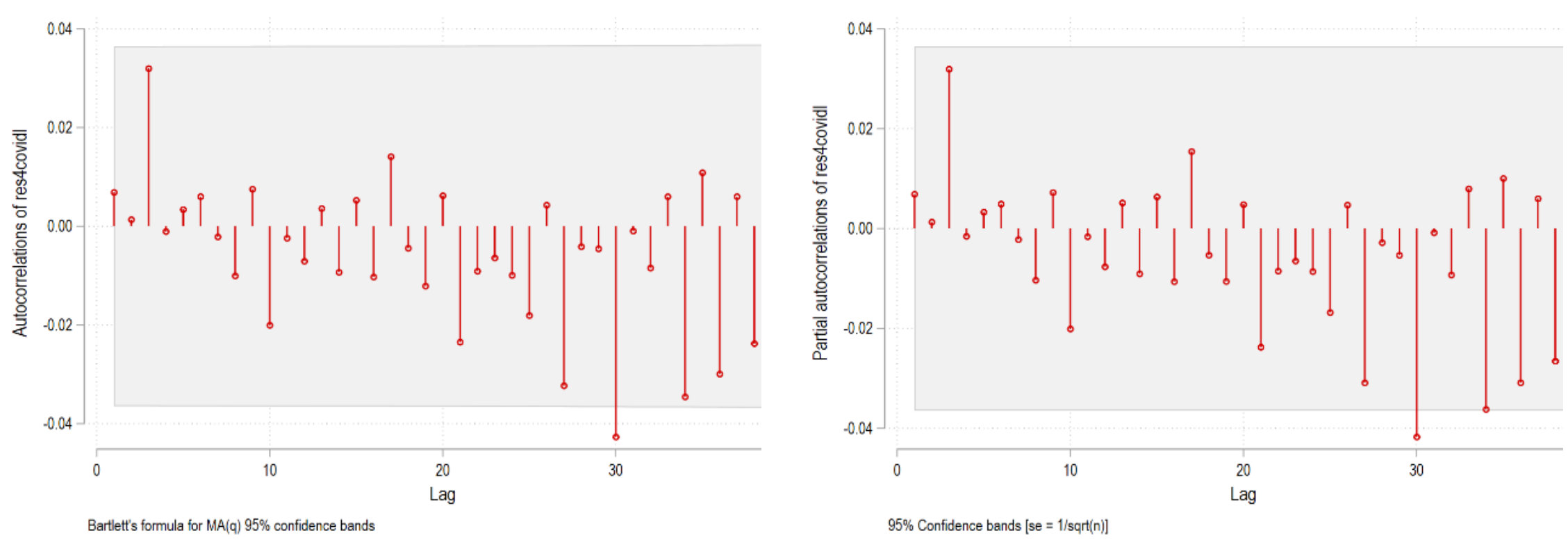

a b

Figure S23. Light Injuries (Speeding): a: AC, b: PAC, City of Quebec

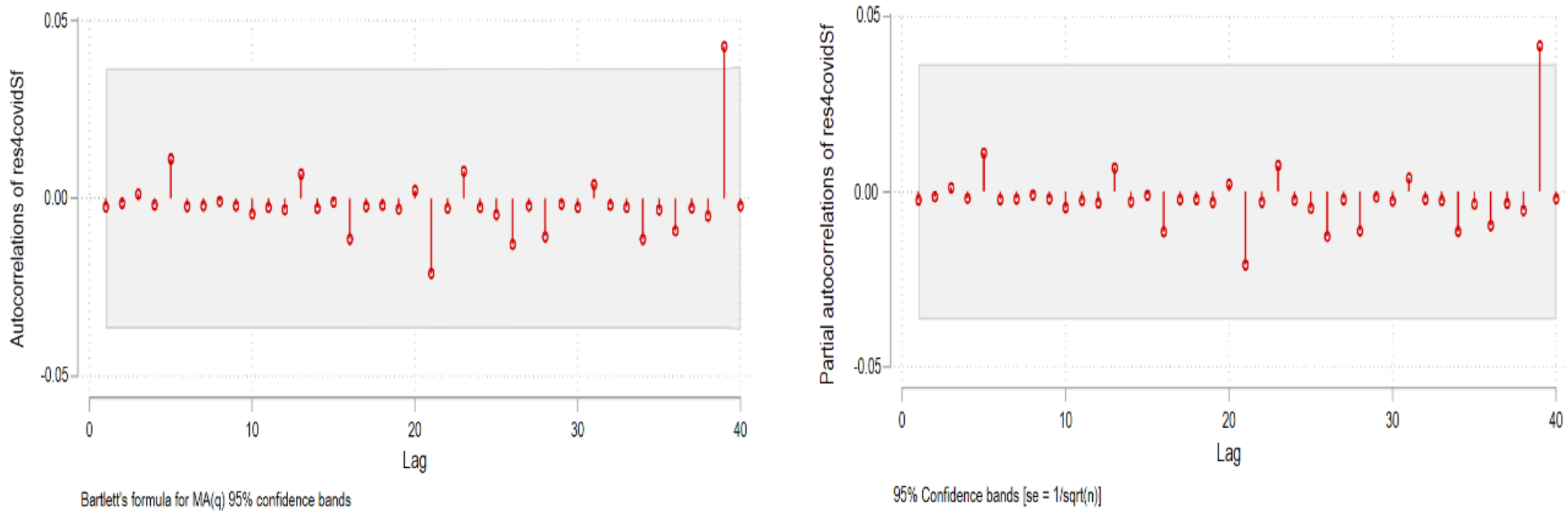

a b

Figure S24. Severe Injuries-Traffic Fatality (Speeding): a: AC, b: PAC, City of Quebec

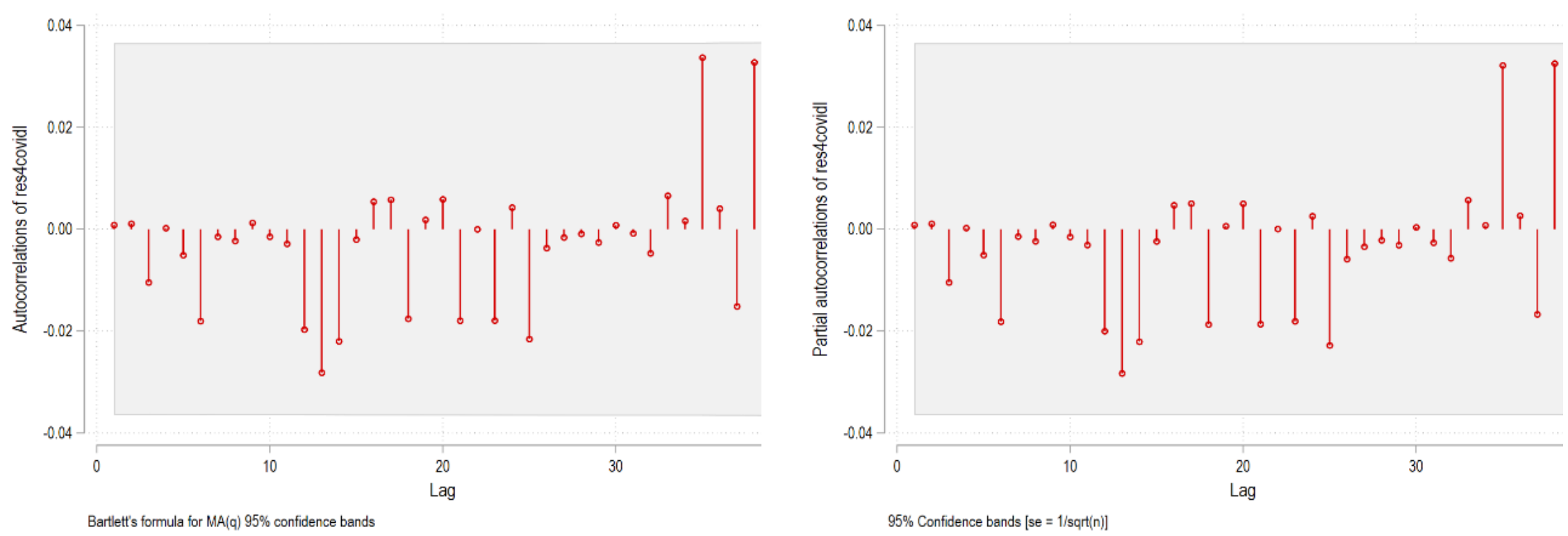

a b

Figure S25. Light Injuries (Speeding): a: AC, b: PAC, City of Laval

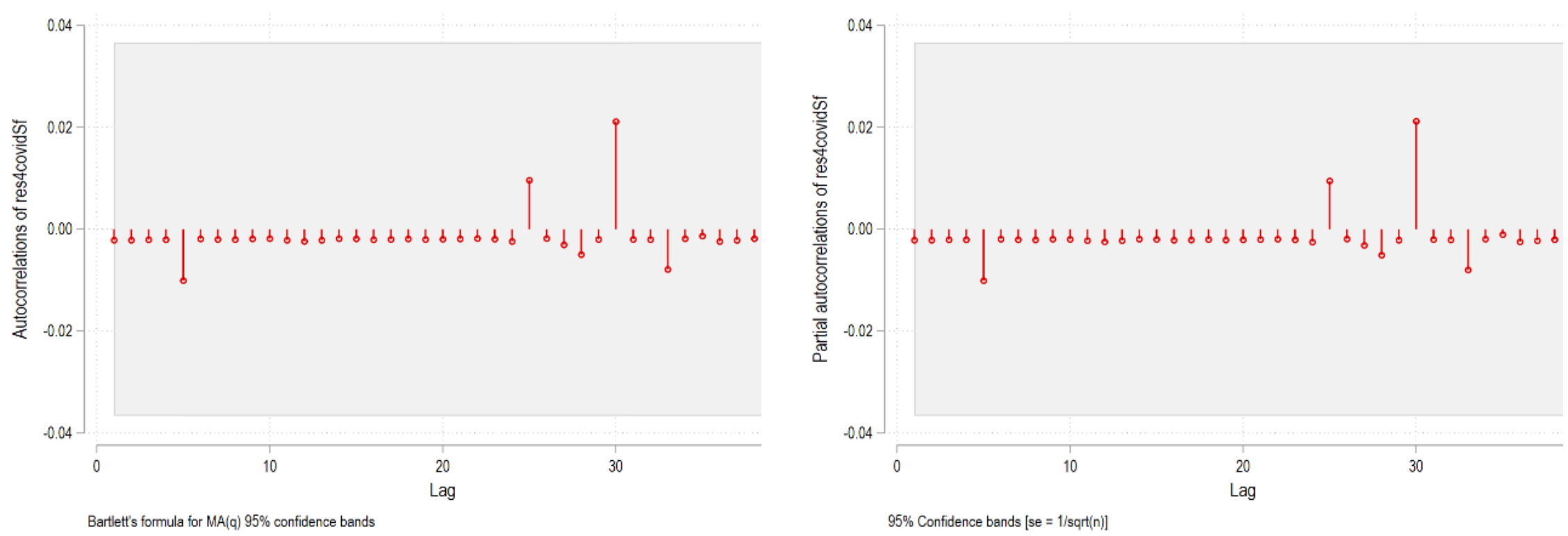

a b

Figure S26. Severe Injuries-Traffic Fatality (Speeding): a: AC, b: PAC, City of Laval

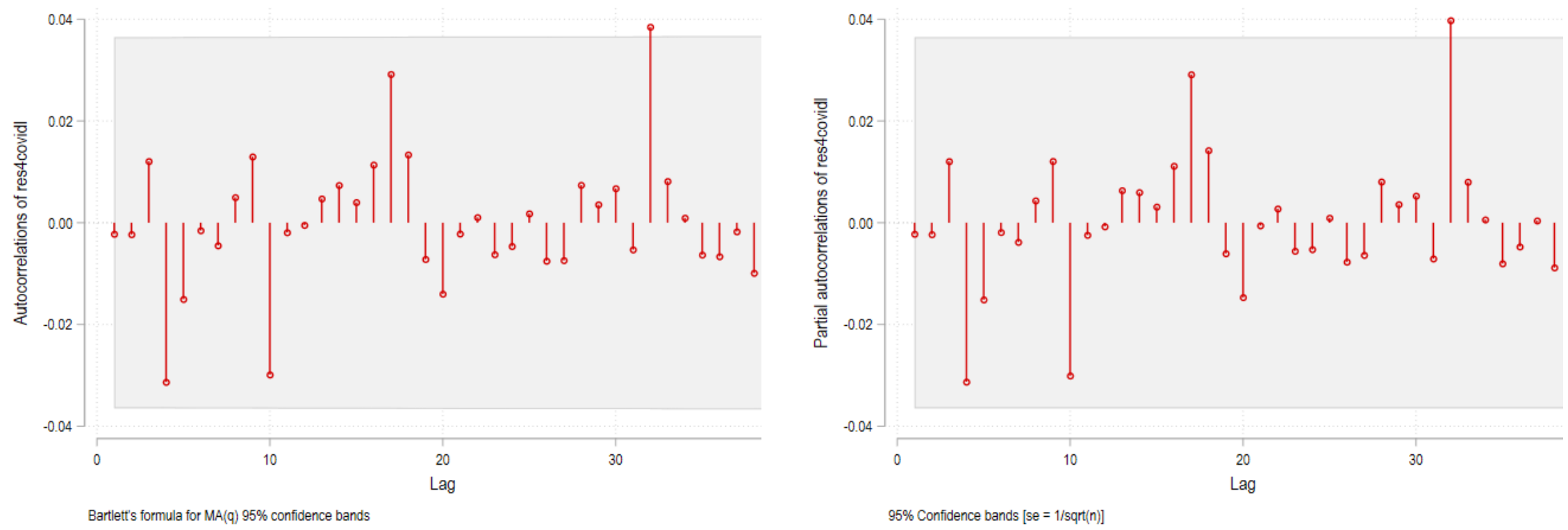

a b

Figure S27. Light Injuries (Speeding): a: AC, b: PAC, City of Longueuil

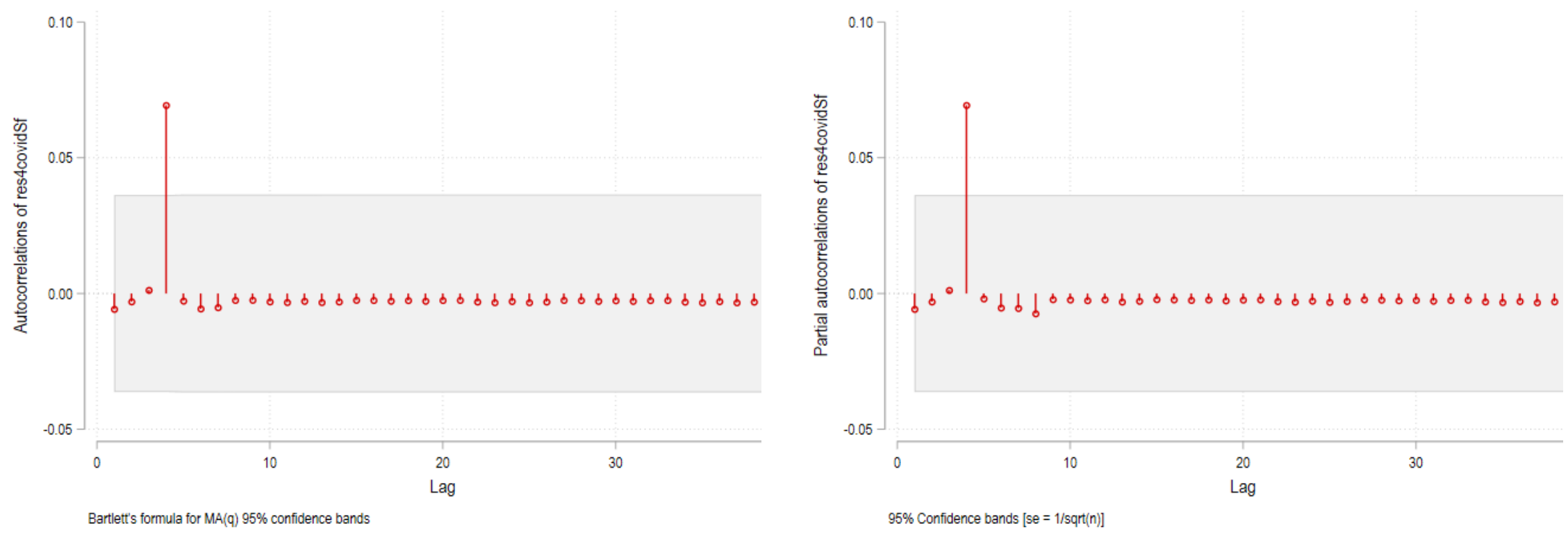

a b

Figure S28. Severe Injuries-Traffic Fatality (Speeding): a: AC, b: PAC, City of Longueuil

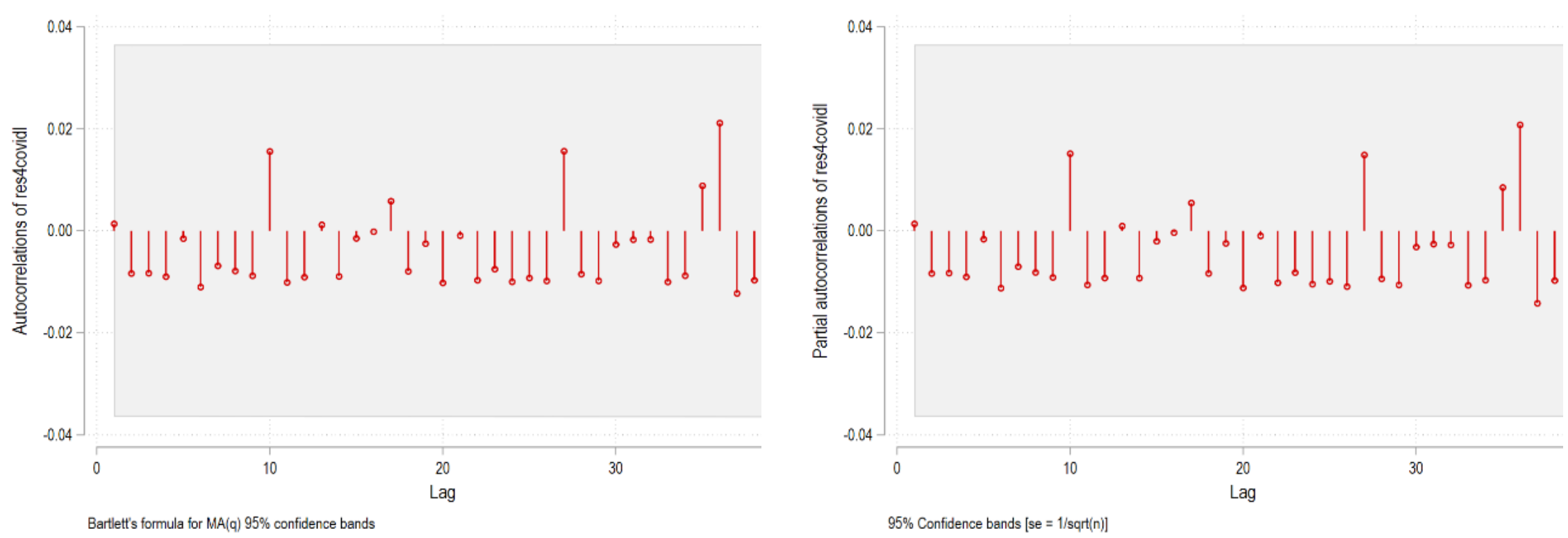

a b

Figure S29. Light Injuries (Speeding): a: AC, b: PAC, City of Sherbrooke

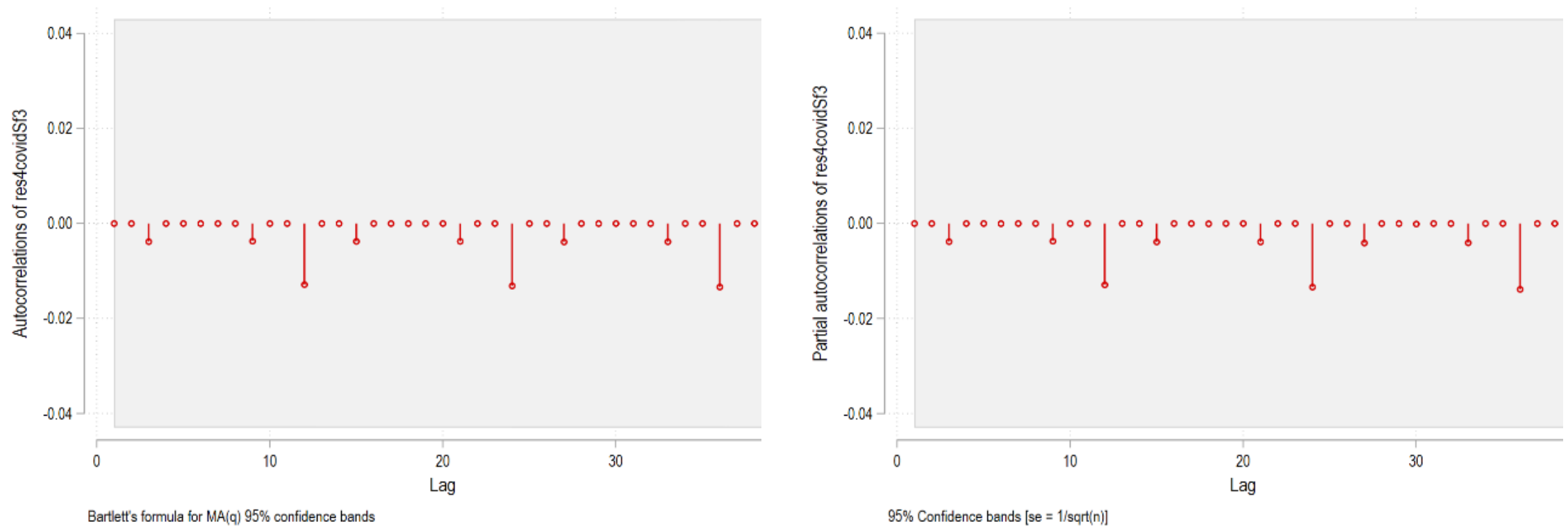

a b

Figure S30. Severe Injuries-Traffic Fatality (Speeding): a: AC, b: PAC, City of Sherbrooke

Table S1. Associations between the QCnPI-Index and alcohol' light injury and KSI rates per 100 000 population, January 1st, 2015 – March 19th, 2022, Zero inflated Poisson models

City	*Outcome*	***Semester 1*** ***March 12th, 2020 - September 11th, 2020***[a]			***Semester 2*** ***September 12st, 2020- March 11th, 2021***[a]			***Semester 3*** ***March 12th, 2021- September 11th, 2021***[a]			***Semester 4*** ***September 12th, 2021- March 11th, 2022***[a]		
		**IRR**	**95% CI**		**IRR**	**95**	**% CI**	**IRR**	**95**	**% CI**	**IRR**	**95**	**% CI**
**Montréal**	Alcohol' light Injuries	**0.80**	**0.70**	**0.90**	**0.74**	**0.65**	**0.86**	0.91	0.80	1.04	0.86	0.64	1.16
	Alcohol' KSI	0.92	0.64	1.33	0.76	0.50	1.17	0.98	0.68	1.40	0.98	0.27	3.57
**Québec City**	Alcohol' light injuries	0.81	0.62	1.04	**0.73**	**0.54**	**0.99**	1.11	0.87	1.40	0.95	0.53	1.74
	Alcohol' KSI	0.64	0.29	1.41	0.99	0.99	1.00	0.58	0.22	1.45	1.01	0.30	3.41
**Laval**	Alcohol' light injuries	0.81	0.61	1.07	0.91	0.68	1.21	0.77	0.52	1.11	1.48	0.75	2.93
	Alcohol' KSI	1.00	0.99	1.00	0.88	0.44	1.76	0.77	0.35	1.67	0.75	0.15	3.80
**Longueuil**	Alcohol' light injuries	**0.64**	**0.49**	**0.84**	0.86	0.70	1.07	1.02	0.66	1.58	0.85	0.48	1.51
	Alcohol' KSI	**0.96**	**0.95**	**0.97**	1.36	0.84	2.18	1.39	0.70	2.75	1.00	0.33	3.06
**Sherbrooke**	Alcohol' light injuries	0.79	0.57	1.10	0.93	0.68	1.29	0.85	0.50	1.45	1.11	0.57	2.14
	Alcohol' KSI	0.95	0.90	1.01	**1.00**	**1.00**	**1.00**	1.00	0.81	1.21	0.99	0.78	1.26

CI: confidence interval; IRR: incidence rate ratios; KSI: Killed or severely injured. Models are adjusted for lags, time trends, daily temperature average, the Cannabis Act, and sine and cosine pair of functions. Bold outcomes indicate $p<0.05$. [a] The QCnPI-Index was rescaled to facilitate the interpretation of the outcomes.

Table S2. Associations between the QCnPI-Index and alcohol' light injury and KSI rates per 100 000 population, January 1st, 2015 – March 19th, 2022, Zero inflated Poisson models without the Cannabis Act

City	*Outcome*	***Semester 1*** ***March 12th, 2020 - September 11th, 2020***[a]			***Semester 2*** ***September 12st, 2020- March 11th, 2021***[a]			***Semester 3*** ***March 12th, 2021- September 11th, 2021***[a]			***Semester 4*** ***September 12th, 2021- March 11th, 2022***[a]		
		**IRR**	**95% CI**		**IRR**	**95**	**% CI**	**IRR**	**95**	**% CI**	**IRR**	**95**	**% CI**
**Montréal**	Alcohol' light Injuries	**0.80**	**0.71**	**0.91**	**0.74**	**0.64**	**0.86**	0.91	0.80	1.04	0.82	0.61	1.09
	Alcohol' KSI	0.87	0.60	1.24	0.75	0.49	1.15	0.97	0.68	1.38	0.98	0.15	6.25
**Québec City**	Alcohol' light injuries	0.81	0.62	1.04	**0.73**	**0.54**	**0.99**	1.10	0.87	1.40	0.95	0.52	1.71
	Alcohol' KSI	0.63	0.29	1.40	0.99	0.99	1.00	0.60	0.25	1.45	1.10	0.35	3.42
**Laval**	Alcohol' light injuries	0.81	0.61	1.07	0.90	0.67	1.20	0.76	0.52	1.11	1.43	0.74	2.79
	Alcohol' KSI	1.00	0.99	1.00	0.88	0.44	1.76	0.77	0.35	1.67	0.78	0.16	3.82
**Longueuil**	Alcohol' light injuries	**0.64**	**0.49**	**0.84**	0.86	0.70	1.06	1.03	0.67	1.58	0.86	0.49	1.54
	Alcohol' KSI	**0.88**	**0.87**	**0.89**	1.32	0.80	2.16	1.34	0.67	2.68	0.88	0.26	3.00
**Sherbrooke**	Alcohol' light injuries	0.80	0.58	1.12	0.92	0.67	1.28	0.83	0.48	1.44	1.03	0.53	1.99
	Alcohol' KSI	0.99	0.99	1.00	1.00	0.99	1.00	0.97	0.93	1.02	0.97	0.91	1.02

CI: confidence interval; IRR: incidence rate ratios; KSI: Killed or severely injured. Models are adjusted for lags, time trends, daily temperature average, and sine and cosine pair of functions. Bold outcomes indicate $p<0.05$. [a] The QCnPI-Index was rescaled to facilitate the interpretation of the outcomes.

Table S3. Associations between the QCnPI and speeding' light injuries and KSI rates per 100 000 population, January 1st, 2015-March 19th, 2022, Zero inflated Poisson models

City	*Outcome*	*Semester 1 March 12th, 2020 - September 11th, 2020*[a]			*Semester 2 September 12st, 2020-March 11th, 2021*[a]			*Semester 3 March 12th, 2021-September 11th, 2021*[a]			*Semester 4 September 12th, 2021-March 11th, 2022*[a]		
		**IRR**	**95**	**% CI**	**IRR**	**95**	**% CI**	**IRR**	**95**	**% CI**	**IRR**	**95**	**% CI**
**Montréal**	Speeding' light injuries	0.88	0.76	1.04	0.87	0.74	1.03	**1.24**	**1.05**	**1.46**	1.12	0.81	1.55
	Speeding' KSI	0.98	0.69	1.40	0.58	0.25	1.33	**1.00**	**1.00**	**1.00**	1.03	0.35	3.05
**Québec City**	Speeding' light injuries	1.00	0.74	1.33	0.52	0.24	1.13	1.09	0.75	1.58	1.07	0.47	2.42
	Speeding' KSI	1.35	0.56	3.24	0.99	0.07	12.68	0.99	0.99	1.00	0.98	0.09	11.39
**Laval**	Speeding' light injuries	0.91	0.60	1.35	0.43	0.17	1.11	0.51	0.22	1.16	1.27	0.62	2.60
	Speeding' KSI	0.96	0.35	2.68	0.99	0.13	7.48	1.00	0.99	1.00	0.99	0.01	111.57
**Longueuil**	Speeding' light injuries	0.84	0.56	1.26	0.89	0.58	1.36	0.97	0.58	1.59	0.96	0.34	2.63
	Speeding' KSI	0.97	0.51	1.82	1.06	0.55	2.03	1.27	0.55	2.91	**0.99**	**0.98**	**0.99**
**Sherbrooke**	Speeding' light injuries	0.96	0.01	125.6	0.99	0.42	2.34	0.99	0.99	1.00	0.99	0.99	1.00
	Speeding' KSI							0.99	0.99	1.00	0.99	0.99	1.00

CI: confidence interval; IRR: incidence rate ratios; KSI: Killed or severely injured. Models are adjusted for lags, time trends, daily temperature average, the Cannabis Act, and sine and cosine pair of functions. Bold outcomes indicate $p<0.05$. [a] The QCnPI was rescaled to facilitate the interpretation of the outcomes.

Table S4. Associations between the QCnPI and speeding' light injuries and KSI rates per 100 000 population, January 1st, 2015-March 19th, 2022, Zero inflated Poisson models without the Cannabis Act.

City	*Outcome*	*Semester 1 March 12th, 2020 - September 11th, 2020*[a]			*Semester 2 September 12st, 2020-March 11th, 2021*[a]			*Semester 3 March 12th, 2021-September 11th, 2021*[a]			*Semester 4 September 12th, 2021-March 11th, 2022*[a]		
		**IRR**	**95**	**% CI**	**IRR**	**95**	**% CI**	**IRR**	**95**	**% CI**	**IRR**	**95**	**% CI**
**Montréal**	Speeding' light injuries	0.90	0.77	1.06	0.86	0.73	1.01	**1.22**	**1.04**	**1.45**	1.03	0.75	1.41
	Speeding' KSI	0.98	0.69	1.40	0.58	0.25	1.33	**1.00**	**1.00**	**1.00**	0.98	0.33	2.87
**Québec City**	Speeding' light injuries	0.98	0.73	1.30	0.52	0.24	1.13	1.10	0.75	1.60	1.13	0.51	2.51
	Speeding' KSI	0.88	0.37	2.09	0.99	0.31	3.17	0.99	0.99	1.00	0.98	0.08	12.52
**Laval**	Speeding' light injuries	0.89	0.60	1.33	0.43	0.17	1.11	0.51	0.22	1.16	1.30	0.64	2.63
	Speeding' KSI	0.93	0.36	2.44	0.99	0.31	3.20	1.00	0.99	1.00	0.98	0.02	40.51
**Longueuil**	Speeding' light injuries	0.79	0.54	1.18	0.87	0.57	1.34	0.96	0.58	1.57	1.06	0.39	2.91
	Speeding' KSI	0.81	0.42	1.55	0.75	0.36	1.54	0.79	0.28	2.21	**0.98**	**0.98**	**0.99**
**Sherbrooke**	Speeding' light injuries	0.96	0.00	1292.27	0.99	0.30	3.24	0.99	0.99	1.00	0.99	0.99	1.00
	Speeding' KSI							0.99	0.99	1.00	1.00	0.99	1.00

CI: confidence interval; IRR: incidence rate ratios; KSI: Killed or severely injured. Models are adjusted for lags, time trends, daily temperature average, and sine and cosine pair of functions. Bold outcomes indicate $p<0.05$. [a] The QCnPI was rescaled to facilitate the interpretation of the outcomes.

Table S5. Associations between the QCnPI-Index and alcohol' light injury and KSI rates per traffic flow, January 1st, 2015 – March 19th, 2022.

City	*Outcome*	***Semester 1*** ***March 12th, 2020 - September 11th, 2020[a]***			***Semester 2*** ***September 12st, 2020- March 11th, 2021[a]***			***Semester 3*** ***March 12th, 2021- September 11th, 2021[a]***			***Semester 4*** ***September 12th, 2021- March 11th, 2022[a]***		
		**IRR**	**95% CI**		**IRR**	**95**	**% CI**	**IRR**	**95**	**% CI**	**IRR**	**95**	**% CI**
**Montréal**	Alcohol' light Injuries	**0.81**	**0.70**	**0.95**	**0.70**	**0.57**	**0.87**	0.88	0.74	1.05	0.83	0.59	1.19
	Alcohol' KSI	0.95	0.60	1.52	0.83	0.48	1.47	1.09	0.73	1.44	0.98	0.32	3.00
**Québec City**	Alcohol' light injuries	0.79	0.59	1.05	0.68	0.44	1.03	1.07	0.83	1.38	0.75	0.36	1.61
	Alcohol' KSI	0.70	0.31	1.58	0.99	0.64	1.52	0.64	0.28	1.47	0.98	0.35	2.73
**Laval**	Alcohol' light injuries	0.87	0.62	1.22	0.80	0.52	1.24	0.82	0.52	1.29	1.26	0.69	2.29
	Alcohol' KSI	0.97	0.63	1.49	0.97	0.63	1.49	0.63	0.19	2.04	0.93	0.16	5.20
**Longueuil**	Alcohol' light injuries	**0.64**	**0.44**	**0.95**	0.92	0.71	1.18	0.90	0.58	1.42	0.65	0.25	1.70
	Alcohol' KSI	0.97	0.63	1.49	1.40	0.84	2.33	1.43	0.71	2.90	0.87	0.14	5.58
**Sherbrooke**	Alcohol' light injuries	0.79	0.53	1.18	0.91	0.60	1.36	0.90	0.47	1.67	0.81	0.29	2.29
	Alcohol' KSI	0.96	0.70	1.31	1.00	0.99	1.00	0.97	0.55	1.70	0.95	0.40	2.24

CI: confidence interval; IRR: incidence rate ratios; KSI: Killed or severely injured. Models are adjusted for lags, time trends, daily temperature average, the Cannabis Act, and sine and cosine pair of functions. The Autocorrelation and partial autocorrelation information, and Ljung-Box and Bartlett tests values for each model are available online in supplementary Figures S11-20 and Table S8 respectively. Bold outcomes indicate $p<0.05$. [a] The QCnPI-Index was rescaled to facilitate the interpretation of the outcomes.

Table S6. Associations between the QCnPI-Index and alcohol' light injury and KSI rates per congestion, January 1st, 2015 – March 19th, 2022.

City	*Outcome*	***Semester 1*** ***March 12th, 2020 - September 11th, 2020[a]***			***Semester 2*** ***September 12st, 2020- March 11th, 2021[a]***			***Semester 3*** ***March 12th, 2021- September 11th, 2021[a]***			***Semester 4*** ***September 12th, 2021- March 11th, 2022[a]***		
		**IRR**	**95% CI**		**IRR**	**95**	**% CI**	**IRR**	**95**	**% CI**	**IRR**	**95**	**% CI**
**Montréal**	Alcohol' light Injuries	**0.83**	**0.72**	**0.97**	**0.73**	**0.59**	**0.91**	0.97	0.82	1.15	0.96	0.68	1.35
	Alcohol' KSI	0.99	0.62	1.56	0.87	0.50	1.53	1.18	0.80	1.75	0.99	0.31	3.03
**Québec City**	Alcohol' light injuries	0.78	0.58	1.04	0.67	0.44	1.00	1.06	0.82	1.37	0.75	0.35	1.59
	Alcohol' KSI	0.70	0.31	1.56	0.99	0.65	1.51	0.63	0.27	1.45	0.98	0.35	2.72
**Laval**	Alcohol' light injuries	0.86	0.61	1.20	0.81	0.53	1.27	0.84	0.54	1.31	1.31	0.73	2.37
	Alcohol' KSI	0.97	0.62	1.50	0.97	0.63	1.49	0.65	0.20	2.10	0.97	0.17	5.55
**Longueuil**	Alcohol' light injuries	**0.65**	**0.44**	**0.96**	0.92	0.71	1.18	0.90	0.58	1.42	0.65	0.25	1.70
	Alcohol' KSI	0.97	0.63	1.49	1.40	0.83	2.33	1.43	0.70	2.90	0.87	0.14	5.58
**Sherbrooke**	Alcohol' light injuries	0.85	0.57	1.26	0.97	0.66	1.46	1.03	0.57	1.90	1.00	0.37	2.73
	Alcohol' KSI	0.96	0.70	1.31	1.00	0.99	1.00	0.97	0.54	1.70	0.95	0.40	2.23

CI: confidence interval; IRR: incidence rate ratios; KSI: Killed or severely injured. Models are adjusted for lags, time trends, daily temperature average, the Cannabis Act, and sine and cosine pair of functions. The Autocorrelation and partial autocorrelation information, and Ljung-Box and Bartlett tests values for each model are available online in supplementary Figures S11-20 and Table S8 respectively. Bold outcomes indicate $p<0.05$. [a] The QCnPI-Index was rescaled to facilitate the interpretation of the outcomes.

Table S7. Associations between the QCnPI-Index and speeding' light injuries and KSI rates per traffic flow, January 1st, 2015- March 19th, 2022.

City	*Outcome*	*Semester 1 March 12th, 2020 - September 11th, 2020[a]*			*Semester 2 September 12st, 2020- March 11th, 2021[a]*			*Semester 3 March 12th, 2021- September 11th, 2021[a]*			*Semester 4 September 12th, 2021- March 11th, 2022[a]*		
		**IRR**	**95**	**% CI**	**IRR**	**95**	**% CI**	**IRR**	**95**	**% CI**	**IRR**	**95**	**% CI**
**Montréal**	Speeding' light injuries	0.93	0.77	1.12	0.86	0.70	1.06	**1.20**	**1.02**	**1.41**	1.29	0.87	1.91
	Speeding' KSI	1.10	0.76	1.49	0.99	0.99	1.00	**1.00**	**1.00**	**1.00**	0.34	0.02	6.69
**Québec City**	Speeding' light injuries	1.08	0.80	1.46	0.59	0.26	1.35	1.07	0.72	1.59	1.23	0.53	2.89
	Speeding' KSI	1.14	0.63	2.08	0.99	0.64	1.55	0.60	0.12	2.95	0.98	0.28	3.51
**Laval**	Speeding' light injuries	0.92	0.67	1.27	0.66	0.41	1.06	0.52	0.19	1.44	1.62	0.86	3.03
	Speeding' KSI	1.59	0.66	3.80	0.99	0.60	1.64	**1.00**	**1.00**	**1.00**	0.98	0.28	3.43
**Longueuil**	Speeding' light injuries	0.91	0.58	1.43	0.99	0.64	1.57	1.00	0.50	2.06	1.20	0.46	3.15
	Speeding' KSI	0.87	0.44	1.72	0.80	0.38	1.69	0.86	0.29	2.61	0.98	0.35	2.77
**Sherbrooke**	Speeding' light injuries	0.97	0.58	1.60	0.99	0.60	1.65	0.81	0.27	2.33	1.26	0.51	3.10
	Speeding' KSI[b]							0.99	0.99	1.00	0.98	0.64	1.49

CI: confidence interval; IRR: incidence rate ratios; KSI: Killed or severely injured. Models are adjusted for lags, time trends, daily temperature average, the Cannabis Act, and sine and cosine pair of functions. The Autocorrelation and partial autocorrelation information, and Ljung-Box and Bartlett tests values for each model are available online in Figures S21-30 and Table S9 respectively. Bold outcomes indicate $p<0.05$. [a] The QCnPI was rescaled to facilitate the interpretation of the outcomes. [b] Speeding KSI incidents were not reported during semesters 1 and 2.

Table S8. Associations between the QCnPI-Index and speeding' light injuries and KSI rates per traffic flow, January 1st, 2015- March 19th, 2022.

City	*Outcome*	*Semester 1 March 12th, 2020 - September 11th, 2020[a]*			*Semester 2 September 12st, 2020- March 11th, 2021[a]*			*Semester 3 March 12th, 2021- September 11th, 2021[a]*			*Semester 4 September 12th, 2021- March 11th, 2022[a]*		
		**IRR**	**95**	**% CI**	**IRR**	**95**	**% CI**	**IRR**	**95**	**% CI**	**IRR**	**95**	**% CI**
**Montréal**	Speeding' light injuries	0.94	0.79	1.13	0.90	0.73	1.11	**1.28**	**1.10**	**1.51**	**1.47**	**1.00**	**2.16**
	Speeding' KSI	1.13	0.79	1.60	0.99	0.99	1.00	**1.00**	**1.00**	**1.00**	0.35	0.02	6.59
**Québec City**	Speeding' light injuries	1.07	0.78	1.45	0.58	0.25	1.33	1.06	0.71	1.58	1.22	0.52	2.86
	Speeding' KSI	1.13	0.62	2.05	0.99	0.65	1.53	0.58	0.11	2.93	0.98	0.29	3.27
**Laval**	Speeding' light injuries	0.93	0.67	1.27	0.67	0.42	1.08	0.54	0.19	1.51	1.67	0.89	3.13
	Speeding' KSI	1.60	0.67	3.81	0.99	0.60	1.65	**1.00**	**1.00**	**1.00**	0.98	0.24	3.91
**Longueuil**	Speeding' light injuries	0.91	0.58	1.43	0.99	0.64	1.57	1.00	0.50	2.05	1.20	0.45	3.15
	Speeding' KSI	0.87	0.44	1.72	0.80	0.38	1.69	0.86	0.29	2.61	0.98	0.35	2.77
**Sherbrooke**	Speeding' light injuries	0.97	0.59	1.59	0.99	0.60	1.64	0.81	0.27	2.33	1.44	0.60	3.50
	Speeding' KSI[b]							0.99	0.99	1.00	0.97	0.65	1.45

CI: confidence interval; IRR: incidence rate ratios; KSI: Killed or severely injured. Models are adjusted for lags, time trends, daily temperature average, the Cannabis Act, and sine and cosine pair of functions. The Autocorrelation and partial autocorrelation information, and Ljung-Box and Bartlett tests values for each model are available online in Figures S21-30 and Table S9 respectively. Bold outcomes indicate $p<0.05$. [a] The QCnPI was rescaled to facilitate the interpretation of the outcomes. [b] Speeding KSI incidents were not reported during semesters 1 and 2.

Table S9. Z scores to assess differences of Incidence Rate Ratios across models per semester for alcohol light injuries and alcohol KSI

City	Outcome	Semester 1 vs Semester 2		Semester 1 vs Semester 3		Semester 1 vs Semester 4		Semester 2 vs Semester 3		Semester 2 vs Semester 4		Semester 3 vs Semester 4	
		**Z**	***p***	**Z**	***p***	**Z**	***p***	**Z**	***p***	**Z**	***p***	**Z**	***p***
**Montréal**	Alcohol' light Injuries	-0.65	0.51	-1.50	0.13	-0.50	0.61	**-2.03**	**0.04**	-0.87	0.38	1.11	0.26
	Alcohol' KSI	0.63	0.52	-0.23	0.81	-0.09	0.92	-0.79	0.42	-0.35	0.72	-0.00	0.99
**Québec City**	Alcohol' light injuries	0.41	0.67	-1.77	0.07	-0.45	0.64	**-2.01**	**0.04**	-0.69	0.48	0.49	0.62
	Alcohol' KSI	-1.09	0.27	0.18	0.85	-0.62	0.53	1.15	0.25	-0.03	0.97	-0.73	0.46
**Laval**	Alcohol' light injuries	-0.56	0.57	0.22	0.83	-1.62	0.10	0.69	0.48	-1.31	0.19	-1.66	0.09
	Alcohol' KSI	0.02	0.98	0.05	0.96	0.04	0.96	0.27	0.78	0.17	0.86	0.01	0.98
**Longueuil**	Alcohol' light injuries	-1.70	0.08	-1.81	0.07	-0.88	0.38	-0.70	0.48	0.03	0.97	0.50	0.61
	Alcohol' KSI	-1.42	0.15	-0.95	0.34	-0.08	0.93	0.02	0.98	0.49	0.43	0.43	0.66
**Sherbrooke**	Alcohol' light injuries	-0.69	0.48	-0.23	0.81	-0.99	0.37	0.28	0.78	-0.47	0.63	-0.61	0.53
	Alcohol' KSI	-1.77	0.08	-0.55	0.58	-0.33	0.73	1.13	0.25	-0.01	0.99	-0.17	0.86

Table S10. Z scores to assess differences of Incidence Rate Ratios across models per semester for speeding light injuries and speeding KSI

City	Outcome	Semester 1 vs Semester 2		Semester 1 vs Semester 3		Semester 1 vs Semester 4		Semester 2 vs Semester 3		Semester 2 vs Semester 4		Semester 3 vs Semester 4	
		**Z**	***p***	**Z**	***p***	**Z**	***p***	**Z**	***p***	**Z**	***p***	**Z**	***p***
**Montréal**	Speeding' light injuries	0.14	0.88	**-2.84**	**0.00**	**-2.00**	**0.04**	**-2.96**	**0.00**	-1.38	0.16	0.52	0.59
	Speeding' KSI	1.15	0.25	-0.06	0.94	-0.80	0.93	-1.20	0.20	-0.82	0.40	-0.06	0.94
**Québec City**	Speeding' light injuries	1.43	0.15	-0.38	0.70	-0.23	0.81	-1.59	0.11	-1.23	0.21	-0.02	0.98
	Speeding' KSI	0.22	0.82	0.75	0.45	0.23	0.81	0.28	0.77	0.00	0.99	-0.28	0.77
**Laval**	Speeding' light injuries	1.39	0.16	1.22	0.22	-0.81	0.41	-0.23	0.81	-1.78	0.07	-1.64	0.10
	Speeding' KSI	-0.01	0.99	-0.00	0.99	0.00	0.99	-0.02	0.97	-0.08	0.93	0.00	0.99
**Longueuil**	Speeding' light injuries	-0.16	0.87	-0.41	0.68	-0.22	0.82	-0.26	0.79	-0.14	0.89	-0.01	0.98
	Speeding' KSI	-0.05	0.95	-0.33	0.73	0.22	0.82	-0.20	0.84	-0.51	0.60	0.61	0.54
**Sherbrooke**	Speeding' light injuries	-0.01	0.99	-0.01	0.99	-0.00	0.99	-0.01	0.98	-0.01	0.98	0.07	0.94
	Speeding' KSI	NA	NA	NA	NA	NA	NA	NA	NA	NA	NA	-1.03	0.30

Table S11. White noise tests on time series of Alcohol-related crashes models

Regions' name	*Semester 1 March2020 to September 2020*				*Semester 2 October 2020 to April 2021*				*Semester 3 May 2021 to October 2021*				*Semester 4 November 2021 to April 2022*			
	Bartlett's (B) statistic	Prob > B	Portmanteau (Q) statistic	Prob > chi2(40)	Bartlett's (B) statistic	Prob > B	Portmanteau (Q) statistic	Prob > chi2(40)	Bartlett's (B) statistic	Prob > B	Portmanteau (Q) statistic	Prob > chi2(40)	Bartlett's (B) statistic	Prob > B	Portmanteau (Q) statistic	Prob > chi2(40)
Montreal																
Light Injuries	0.24	1.00	3.06	1.00	0.24	1.00	2.83	1.00	0.34	0.99	2.57	0.99	0.33	0.99	3.18	1.00
Severe Injuries- Traffic Fatality	0.52	0.95	28.78	0.90	0.54	0.93	28.13	0.92	0.52	0.94	28.59	0.91	0.54	0.93	28.31	0.91
Quebec																
Light Injuries	0.28	1.00	5.04	1.00	0.35	0.98	4.71	1.00	3.21	0.98	5.14	1.00	2.64	1.00	4.20	1.00
Severe Injuries- Traffic Fatality	0.24	0.99	6.13	1.00	0.27	1.00	6.81	1.00	2.24	1.00	30.63	0.85	3.01	0.96	6.65	1.00
Laval																
Light Injuries	0.23	1.00	1.02	0.87	0.22	1.00	1.11	0.76	0.18	1.00	1.01	0.75	0.18	1.00	0.76	0.68
Severe Injuries- Traffic Fatality	0.30	1.00	14.39	0.99	0.33	0.99	13.72	0.98	0.29	1.00	12.80	1.00	0.31	1.00	12.03	1.00
Longueuil																
Light Injuries	0.23	1.00	0.78	1.00	0.29	1.00	0.80	1.00	0.25	1.00	0.80	1.00	0.24	1.00	0.92	1.00
Severe Injuries- Traffic Fatality	0.34	0.99	2.93	0.99	0.31	1.00	15.31	1.00	0.31	1.00	21.15	0.99	0.30	1.00	2.87	1.00
Sherbrooke																
Light Injuries	0.20	1.00	1.75	1.00	0.19	1.00	1.76	1.00	0.19	1.00	1.70	1.00	0.26	1.00	2.78	1.00
Severe Injuries- Traffic Fatality	0.33	0.99	2.93	1.00	0.34	0.99	1.54	1.00	0.31	1.00	2.93	1.00	0.31	1.00	2.79	1.00

Table S12. White noise tests on time series of speeding-related crashes models

Regions' name	*Semester 1 March2020 to September 2020*				*Semester 2 October 2020 to April 2021*				*Semester 3 May 2021 to October 2021*				*Semester 4 November 2021 to April 2022*			
	Bartlett's (B) statistic	Prob > B	Portmanteau (Q) statistic	Prob > chi2(40)	Bartlett's (B) statistic	Prob > B	Portmanteau (Q) statistic	Prob > chi2(40)	Bartlett's (B) statistic	Prob > B	Portmanteau (Q) statistic	Prob > chi2(40)	Bartlett's (B) statistic	Prob > B	Portmanteau (Q) statistic	Prob > chi2(40)
Montreal																
Light Injuries	0.21	1.00	6.15	1.00	0.27	1.00	7.90	1.00	0.57	0.90	6.66	1.00	0.22	1.00	7.40	1.00
Severe Injuries- Traffic Fatality	0.31	1.00	24.39	0.97	0.38	0.99	25.92	0.95	1.09	0.18	17.62	0.99	0.40	0.99	12.80	1.00
Quebec																
Light Injuries	0.21	0.99	13.16	1.00	0.51	0.99	11.22	1.00	0.51	0.98	11.67	1.00	0.45	0.99	10.90	1.00
Severe Injuries- Traffic Fatality	0.26	1.00	33.03	0.77	0.22	1.00	42.37	0.36	0.22	1.00	39.74	0.48	0.23	1.00	4.87	1.00
Laval																
Light Injuries	0.55	0.91	18.64	0.99	0.64	0.80	27.74	0.92	0.65	0.79	28.39	0.91	0.70	0.71	27.96	0.92
Severe Injuries- Traffic Fatality	0.18	1.00	10.83	1.00	0.15	1.00	11.82	1.00	0.13	1.00	25.25	0.96	0.15	1.00	11.55	1.00
Longueuil																
Light Injuries	0.45	0.98	13.76	1.00	0.49	0.97	13.08	1.00	0.57	0.89	15.18	0.99	0.50	0.96	12.10	1.00
Severe Injuries- Traffic Fatality	0.80	0.53	22.12	0.99	0.82	0.51	22.09	0.99	0.78	0.57	29.80	0.88	0.81	0.52	26.76	0.94
Sherbrooke																
Light Injuries	0.29	1.00	5.62	1.00	0.27	1.00	5.51	1.00	0.31	1.00	6.03	1.00	0.40	0.99	7.60	1.00
Severe Injuries- Traffic Fatality	0.21	0.98	7.53	0.1.00	0.22	0.99	8.03	1.00	0.53	1.00	6.77	0.99	0.51	1.00	8.50	1.00

Table S13. Population values per city per year

**City**	**Year**							
	**2015**	**2016**	**2017**	**2018**	**2019**	**2020**	**2021**	**2022**
Montréal	1704694	1710519	1722170	1727996	1745472	1751298	1757123	1762949
Québec City	531902	533657	537169	538924	544191	545947	547703	549459
Laval	421393	423572	427932	430112	436652	438832	441012	443192
Longueuil	238897	240455	243572	245131	249807	251365	252924	254483
Sherbrooke	161323	162485	164811	165973	169461	170624	171787	172950
